# Tetrahydrobenzimidazole TMQ0153 triggers apoptosis, autophagy and necroptosis crosstalk in chronic myeloid leukemia

**DOI:** 10.1038/s41419-020-2304-8

**Published:** 2020-02-07

**Authors:** Sungmi Song, Jin-Young Lee, Ludmila Ermolenko, Aloran Mazumder, Seungwon Ji, Heeju Ryu, HyeJin Kim, Dong-Wook Kim, Jung Weon Lee, Mario Dicato, Christo Christov, Michael Schnekenburger, Claudia Cerella, Déborah Gérard, Barbora Orlikova-Boyer, Ali Al-Mourabit, Marc Diederich

**Affiliations:** 1 College of Pharmacy, Seoul National University, 1 Gwanak-ro, Gwanak-gu, Seoul, 08626 Korea; 2 Département SNCM (Substances Naturelles et Chimie Médicinale), ICSN-CNRS, LabEx LERMIT, Centre de Recherche de Gif-sur-Yvette, Avenue de la Terrasse (Bat. 27), 91190 Gif-sur-Yvette, France; 3 Catholic University, Seoul St. Mary’s Hospital, Banpo dong 505, Seocho Gu, Seoul, Korea; 4 Laboratoire de Biologie Moléculaire du Cancer, Hôpital Kirchberg, 9, rue Edward Steichen, L-2540 Luxembourg, Luxembourg; 5 Service d’Histologie, Faculté de Médicine, Université de Lorraine, and INSERM U1256 NGERE, 54000 Nancy, France

**Keywords:** Chronic myeloid leukaemia, Chronic myeloid leukaemia

## Abstract

By comparing imatinib-sensitive and -resistant chronic myeloid leukemia (CML) cell models, we investigated the molecular mechanisms by which tetrahydrobenzimidazole derivative TMQ0153 triggered caspase-dependent apoptosis at low concentrations accompanied by loss of mitochondrial membrane potential (MMP) and increase of cytosolic free Ca^2+^ levels. Interestingly, at higher concentrations, TMQ0153 induced necroptotic cell death with accumulation of ROS, both preventable by *N*-acetyl-L-cysteine (NAC) pretreatment. At necroptosis-inducing concentrations, we observed increased ROS and decreased ATP and GSH levels, concomitant with protective autophagy induction. Inhibitors such as bafilomycin A1 (baf-A1) and siRNA against beclin 1 abrogated autophagy, sensitized CML cells against TMQ0153 and enhanced necroptotic cell death. Importantly, TMQ153-induced necrosis led to cell surface exposure of calreticulin (CRT) and ERp57 as well as the release of extracellular ATP and high mobility group box (HMGB1) demonstrating the capacity of this compound to release immunogenic cell death (ICD) markers. We validated the anti-cancer potential of TMQ0153 by in vivo inhibition of K562 microtumor formation in zebrafish. Taken together, our findings provide evidence that cellular stress and redox modulation by TMQ0153 concentration-dependently leads to different cell death modalities including controlled necrosis in CML cell models.

## Introduction

Imatinib kills leukemic cells essentially via apoptosis but triggers primary or secondary resistance in approximately 20–30% of patients^1^. Second-generation tyrosine kinase inhibitors (TKIs) such as dasatinib and nilotinib re-activate apoptotic cell death induction^2,3^ in patients with imatinib resistance, however, de novo resistance against these TKIs was also reported^4^.

Pharmacological agents that target BCR-ABL-independent molecular targets in CML by initiating non-apoptotic cell death may overcome both BCR-ABL- and apoptosis-related resistance mechanisms by targeting unrelated vulnerabilities of CML cells specifically related to oxidative and metabolic stress metabolism. Transformation of leukemic cells by Bcr-Abl is associated with metabolic alterations and increased reactive oxygen species (ROS) generation. In addition, ROS levels are tightly regulated in normal hematopoiesis but are chronically elevated in CML. Koptyra et al. showed that Bcr-Abl kinase stimulates ROS that cause oxidative DNA damage that results in the mutation of the kinase domain, leading to imatinib resistance. In addition, Landry et al.^5^ published that NADPH oxidase (Nox) activity affects intracellular ROS levels in Bcr-Abl positive cells, enhancing survival signaling. Therefore, targeting the altered metabolism and accumulation of ROS in CML cells could be of therapeutic interest as exacerbation of intracellular ROS levels constitute one of the main mechanisms of most chemo- and radio-therapeutic agents, eventually killing cells whether by apoptosis or programmed necrosis, depending on the metabolic status of the cell. Apoptosis and necroptosis are major cell death mechanisms that result in opposite immune responses^6^. Whereas apoptotic cell death was described to trigger immunotolerant responses, necroptotic cell death releases molecules that are related to inflammation and activate immune responses as implicated to ICD^7^. ICD is also triggered by stress response mechanisms such as autophagy, ROS, and endoplasmic reticulum (ER) stress and unfolded protein response (UPR)^8^. These stress responses further lead to cell death and DAMP release that are required for the activation of anticancer immune response by elevating immunogenicity of dying cells via ICD^9^.

Controlled necrosis pathways cause a disequilibrium of the redox metabolome leading to depletion of ATP and glutathione (GSH) eventually triggering an energetic catastrophe. The crosstalk between the redox metabolism, autophagy and necroptosis offers an interesting therapeutic target in CML. Under oxidative and metabolic stress, autophagy becomes a cellular homeostasis mechanism that aims to re-establish the cellular energy balance, among others^10^ allowing cell survival under stress conditions. Moreover, autophagy cross-talks with apoptosis at the level of caspase-8 degradation as well as with non-apoptotic or necrotic programmed cell death mechanisms^11^.

We previously reported the synthesis of various tetrahydrobenzimidazole derivatives and investigated their cytotoxic potential against hematopoietic cancer cell lines. Among these derivatives, the quinone TMQ0153 exhibited significant differential cytotoxicity against cancer cells^12^. The usefulness of such quinones as inducers of non-canonical cell death in CML remains to be investigated.

## Materials and methods

### Compounds

Tetrahydrobenzimidazole (TMQ) 0153 was synthetized from p-benzoquinone as previously described^12^. 2′-deoxy-5-azacytidine (5-aza; A3656), 3-methyladenine (3-MA; M9281), bafilomycin (baf-A1; #B1793), N-acetyl-L-cysteine (NAC; LAA21), thapsigargin (TSG; T9033), PP242 (P0037), shikonin (SHK; S7576) and necrostatin-1 (Nec-1; N9037) were purchased from Sigma-Aldrich (St. Louis, MO, USA). Chloroquine (CQ; NZ-51031-K200) was purchased from Enzo Life Science (ENZ-51031-0050). Tiron (SC-253669), Trolox (SC-200810), buthionine sulfoximine (BSO) (SC-200824) were obtained from Santa-Cruz Biotechnology (CA, USA). Hydrogen peroxide (H_2_O_2_) was purchased from Junsei Chemical (23150-0350) (Tokyo, Japan).

### Cell culture

Chronic myeloid leukemia cell lines K562, KBM-5, and MEG01 were cultured in RPMI 1640 medium (Lonza, Walkersville, MD, USA) supplemented with 10% (v/v) fetal calf serum (FCS; Biowest, Riverside, MO, USA) and 1% (v/v) antibiotic–antimycotics (Lonza, Walkersville, MD, USA) at 37 °C and 5% of CO_2_. KBM-5 cells were kindly donated by Dr. Bharat B. Aggarwal. Imatinib-resistant KBM5-T315I cells (KBM5R) cells were obtained by sequentially increasing the concentration of imatinib from 0.25, to 1 µM imatinib in IMDM media supplemented with 10% (v/v) fetal calf serum and 1% (v/v) antibiotic–antimycotics^13^. Imatinib-resistant K562 (K562R) cells were a gift of the Catholic University of Seoul and cultured in RPMI 1640 medium with 25 mM HEPES (Lonza) supplemented with 10% (v/v) FCS and 1% (v/v) antibiotic–antimycotics. Both resistant cell types were cultured with 1 µM of imatinib and washed three times before each experiment. Lung carcinoma A549 and breast adenocarcinoma MCF7 cells were obtained from the American Type Culture Collection (ATCC, Manassas, USA) were cultured in RPMI 1640 medium (Lonza, Walkersville, MD, USA) supplemented with 10% (v/v) fetal calf serum (FCS; Biowest, Riverside, MO, USA) and 1% (v/v) antibiotic–antimycotics (Lonza, Walkersville, MD, USA). Normal B lymphocyte RPMI-1788 from the Korean cell line Bank (KCLB, Seoul, South Korea) were cultured in RPMI 1640 medium (Lonza, Walkersville, MD, USA) supplemented with 10% (v/v) fetal calf serum (FCS; Biowest, Riverside, MO, USA) and 1% (v/v) antibiotic–antimycotics (Lonza, Walkersville, MD, USA) at 37 °C and 5% of CO_2_. All cells were cultured according to standard procedures. Mycoplasma testing was done bi-monthly. STR profiling was done bi-annually.

Peripheral blood mononuclear cells (PBMCs) were isolated by density gradient centrifugation using Ficoll-Hypaque (GE Healthcare, Roosendaal, The Netherlands) from freshly collected buffy coats as previously described^13,14^, obtained from healthy adult human volunteers (Red Cross, Luxembourg, Luxembourg) after ethical approval as well as written informed consent from each volunteer. After isolation, cells were incubated overnight at 2 × 10^6^ cells/mL in RPMI 1640 (supplemented with 1% antibiotic–antimycotic and 10% FCS (BioWhittaker, Verviers, Belgium) at 37 °C and 5% CO_2_ in a humidified atmosphere. The day after, cell concentration was adjusted at 1 × 10^6^ cells/mL using the same fresh complete medium and then treated as indicated.

### Cell viability and cell death assessment

A Trypan blue exclusion assay (Lonza) was used to assess cell viability and IC_50_ values were also calculated on data obtained from Trypan blue assay. The mode of cell death was determined and quantified after determination of the nuclear morphology was evaluated under fluorescence microscopy (Nikon Eclipse Ti-U, Nikon Instruments Korea, South Korea) after cell staining with 1 µg/mL Hoechst 33342 (Sigma-Aldrich,) and 1 μg/mL propidium iodide staining (Sigma-Aldrich). Caspase 3/7 activity was assessed by Caspase-Glo 3/7 Assay (Promega, Madison, WI, USA), and intracellular ATP levels were measured using the CellTiter-Glo Luminescent Cell Viability Assay (Promega, Madison, WI, USA).

### Colony formation assays

Colony formation assays were performed as previously published^15^.

### Protein extraction and western blotting

Whole-cell extracts were prepared using M-PER^®^ (Thermofisher, R7007, Waltham, MA, USA) supplemented by 1× protease inhibitor cocktail (Complete EDTA-free; Roche, Basal, Switzerland) according to the manufacturer’s instructions. Western blots were performed using the following primary antibodies: anti-caspase 7 (9494S), anti-caspase 9 (9502S), anti-caspase 8 (9746), anti-PARP-1 (9542), anti-Mcl-1 (4572S), anti-LC3B (2775), anti-p62 (5114), anti-Beclin 1 (3738) and anti-RIP3 (#13526) from Cell Signaling (Danvers, MA, USA); anti-caspase 3 (sc-56053), anti-PARP-1 (C2-10; sc-53643) from Santa Cruz Biotechnology (CA, USA); anti-Bcl-xL (610212), anti-RIP1 (610458) from BD Pharmingen (San Jose, CA, USA); anti-beta actin (5441) from Sigma Aldrich. Bands were quantified using Image Quant TL (GE Healthcare, Pittsburgh, PA, USA).

### Morphology analysis

For Giemsa staining, cells were spun onto a glass slide for 5 min at 800 × *g* using a cytopad with caps (ELITech Biomedical Systems, USA). Cells were then fixed and stained with the Diff-Quik staining kit (Dade Behring S.A., USA) according to the manufacturer’s protocol and pictures were taken under a microscope (Nikon Eclipse Ti-U, Nikon Instruments Korea, South Korea). A total of 50 cells were counted in one area, and three independent areas were counted for each set of three independent experiments.

### Transmission electron microscopy

For transmission electron microscopy (TEM), 5 × 10^6^ cells were pelleted and fixed in 2.5% glutaraldehyde (Electron Microscopy Sciences, USA) diluted in 0.1 M sodium cacodylate buffer, pH 7.2 (Electron Microscopy Sciences, USA) overnight. Cells were then rinsed with sodium cacodylate buffer twice and post-fixed in 2% osmium tetroxide for 2 h at room temperature. Samples were washed with distilled water and then stained with 0.5% uranyl acetate at 4 °C for overnight. After 24 h, samples were dehydrated through a graded series of ethanol solutions to water followed by propylene oxide, and then infiltrated in 1:1 propylene oxide/Spurr’s resin. Samples were kept overnight embedded in Spurr’s resin, mounted in molds and left to polymerize in an oven at 56 °C for 48 h. Ultrathin sections (70–90 nm) were obtained with ultramicrotome, EM UC7 (Leica, Germany). Sections were stained with uranyl acetate and lead citrate and subsequently examined with a JEM1010 transmission electron microscope (JEOL, Japan).

### Analyses of autophagic vesicles

For fluorescence microscopy analysis, 3 × 10^6^ cells were stained with Cyto-ID® Green dye and Hoechst 33342, according to manufacturer’s instructions (Enzo Life Science). Cells were observed by confocal microscopy (Leica TCS SP8, Germany). Segmentation of objects of interest was based on 31 parameters assessing color, texture and edge and was carried out in Ilastik, version 1.3.0 (https://www.ilastik.org/), developed by the European Molecular Biology Laboratory, Heidelberg^16^. Classifiers trained for these parameters on a set of representative images were then applied to batch process multiple images as described in Ilastik’s user manual. Binary masks thus obtained were measured in FIJI^17^ after applying a size filter to remove small size artefacts resulting from segmentation. Results were compared by Kruskal–Wallis test followed by Conover post-test further adjusted by the Benjamini-Hochberg FDR method (www.astatsa.com). Overall, the number of images evaluated in the different groups was as follows: control group *n* = 7, 4 h group *n* = 7, 8 h group *n* = 6, and PP242 treated group *n* = 5.

### Measurement of cytosolic calcium levels

Experiments were based on published procedures with modifications^18^. Cells were stained with 500 nM Fluo-3-AM (Thermo Fisher, R7007, Waltham, MA, USA) for 25 min at 37 °C. After 15 min at room temperature, cytosolic Ca^2+^ levels were assessed by flow cytometry (FACS Calibur, Becton Dickinson, San Jose, CA, USA) and data were recorded statistically (10,000 events/sample) using the CellQuest Pro software (BD, Biosciences). Data were analyzed using the Flow-Jo 8.8.7 software (Tree Star, Inc., Ashland, OR, USA) and results were expressed as mean fluorescence intensity (MFI).

### Determination of the oxygen consumption rate

The oxygen consumption rate (OCR) was measured using a Seahorse XFp cell mito stress Assay (#103010-100, Agilent, USA) ran on a Seahorse XFp analyzer (Agilent, Yongsan-gu, Seoul) according to manufacturer’s instructions. Briefly, cells were seeded at 200,000 cells per well and treated with TMQ0153 for 4 h in 175 μL medium. Before measurements, plates were equilibrated in a CO_2_-free incubator at 37 °C for 1 h. Analysis were performed using 1.5 µM oligomycin, 0.5 µM carbonyl cyanide-4-(trifluoromethoxy)phenylhydrazone (FCCP), and 1 µM rotenone/antimycin A as indicated. Data were analyzed using the Seahorse XF Cell Mito stress rest report generator software (Agilent).

### Transfections

Cells were transfected with 4.5 µL HiPerFect Transfection reagent (HPF; Qiagen, Germantown, MD, USA,) and 5 nM small interfering RNAs (siRNAs; Qiagen) targeting the human beclin 1 (BCN-1) gene [NM003766; SiBEC-1_1: Hs_BECN1_1 (SI00055573) and 10 nM SiBEC-1_2: Hs_BECN1_2 (SI00055580)] or non-targeting (AllStars Negative Control siRNA) as described elsewhere^19^. 24 h post-transfection, medium was replaced, and cells were treated as indicated on figures.

### Analysis of ROS, mitochondrial membrane potential, mitochondrial and lysosomal membrane mass

ROS levels were probed by using 10 μM on 2-7-dichlorodihydrofluorescein diacetate (H_2_DCF-DA; Life Technologies, Carlsbad, USA) and analyzed by flow cytometry as previously described^20^. To monitor lysosomal mass, mitochondrial membrane potential, and mitochondrial mass, cells were incubated at 37 °C for 30 min with 20 nM LysoTracker Red DND-99, 50 nM MitoTracker Red CMXRos (all from Molecular Probes, Invitrogen, Grand Island, NY, USA), respectively, and then analyzed by flow cytometry. Data were recorded statistically (10,000 events/sample) using the CellQuest Pro software. Data were analyzed using the Flow-Jo 8.8.7 software and results were expressed as mean fluorescence intensity (MFI).

### Glutathione measurements

Reduced (GSH) and oxidized (GSSG) glutathione measurements were performed using the GSH/GSSG-Glo™ Assay kit (Promega, Madison, WI, USA) following the manufacturer’s instructions. The luminescence signal was acquired using with a microplate luminometer Centro LB 960 and data were recorded using the MikroWin 2000 software package (Berthold Technologies, Bad Wildbad, Germany).

### Quantification of HMGB1 release

Quantification of HMGB1 release in cell culture supernatants was assessed by enzyme-linked immunosorbent assay kit from Shino-Test-Corporation (Jinbocho, Chiyoda-ku, Tokyo, Japan) according to the manufacturer’s instructions. Absorbance data were collected using with a SpectraMax i3x microplate reader and data were recorded using the SoftMax Pro 7.0 software package (Sunnyvale, California, USA).

### Measurement of extracellular ATP content

Extracellular ATP levels in the supernatant were assessed by the ENLITEN® ATP Assay system bioluminescence detection kit (Promega, Madison, WI, USA) following the manufacturer’s protocol. Luminescence signal was acquired with a microplate luminometer Centro LB 960 and data were recorded using the MikroWin 2000 software package (Berthold Technologies, Bad Wildbad, Germany).

### Analysis of calreticulin and ERp57 exposure

Cells were collected, washed twice with 1x PBS and fixed in 0.25% paraformaldehyde in 1x PBS at 4 °C. After 5 min incubation, cells we washed twice in cold 1× PBS and incubated for 30 min at RT with anti-calreticulin (CRT; Ab2907, Abcam, Cambridge, UK) or ERp57 (Ab10287, Abcam) primary antibody diluted (1:50) in cold blocking buffer (2% FCS in 1× PBS) and then incubated for 30 min with an Alexa488-conjugated monoclonal secondary antibody (A11034) diluted (1:50) in blocking buffer. Isotype-matched Alexa488-conjugated IgG antibodies were used as a control. Samples were then analyzed by flow cytometry. Data were recorded statistically (10,000 events/sample) using the CellQuest Pro software and analyzed using the Flow-Jo 8.8.7 software Results were expressed as mean fluorescence intensity (MFI). Samples were also analyzed by fluorescence microscopy (Nikon Eclipse Ti-U, Nikon Instruments Korea, South Korea).

### Zebrafish toxicity assays and cancer cell xenografts

Cancer xenograft assays were performed as previously published^21^. Briefly, 200 K562 cells were stained for 2 h by 4 µM of Cell tracker CM-Dil dye (Invitrogen, Grand Island, NY, USA), then treated with TMQ0153 at indicated concentrations for 8 h and injected as described. Fertilized zebrafish eggs were used up to 5 days post fertilization. Sample size for toxicity assays was 10 zebrafish (*Danio rerio*) per condition. Sample size for microinjection assays was 9 zebrafish per condition. No zebrafish were excluded from the study. Experiments were performed in agreement with the animal research committee of the College of Pharmacy of Seoul National University.

### In silico drug-likeness properties

In silico drug-likeness properties according to Lipinski’s ‘rule-of-five’ and other parameters for drug-likeness and oral bioavailability were evaluated by using the SCFBio website (www.scfbio-iitd.res.in/).

### Bioinformatics analysis

The Microarray Innovations in Leukemia (MILE) dataset^22,23^ (GSE13159) was downloaded from the Gene Expression Omnibus repository^22^ and normalized using the Robust Multichip Average (RMA) algorithm from the affy R package (version 1.62.0)^24^. Boxplot was generated using the *ggboxplot* function of the ggpubr R package (version 0.2.2) in R 3.6.0^25^ and RStudio^26^.

### Statistical analysis

All experiments are randomized and blinded. Block randomization was used to randomize samples/zebra fish larvae into groups of indicated sample size. No zebrafish were excluded from analysis. Data are expressed as the mean ± S.D. and significance was estimated by using one-way or two-way ANOVA tests using Prism 8 software, GraphPad Software (La Jolla, CA, USA). Statistical significances were evaluated at *p*-values below 0.05 and represented by the following legend: *p ≤ 0.05, **p ≤ 0.01, ***p ≤ 0.001; post hoc analyses Dunnett; Sidak; Tukey). All histograms represent the mean ± SD of at least three independent experiments. Statistical power analysis was used to ensure adequate sample size for detecting significant difference between samples. The variance is similar between groups that are being statistically compared.

## Results

### Cellular uptake and drug-likeness potential of TMQ0153 in human leukemia cells

We first assessed the uptake of the auto-fluorescent compound TMQ0153^12^ by K562 cells using flow cytometry after 8, 24, 48, and 72 h of treatment with concentrations up to 50 µM. Auto-fluorescence was detected in all three channels (FL1-530/30 nm, FL2-585/42 nm, and FL3-670LP) up to 72 h (Supplementary Fig. 1A) and TMQ0153 (50 µM) was detected by confocal microscopy after 24 h of treatment when cell viability was 25% (Supplementary Fig. 1B), validating the time- and dose-dependent uptake of TMQ153. Moreover, TMQ153 follows Lipinski’s ‘rule of five’ (Supplementary Table I), confirming its potential drug-likeness properties.

### TMQ0153 inhibits cell viability, proliferation and colony formation capacity of drug-sensitive and -resistant cancer cells

**Fig. 1:**
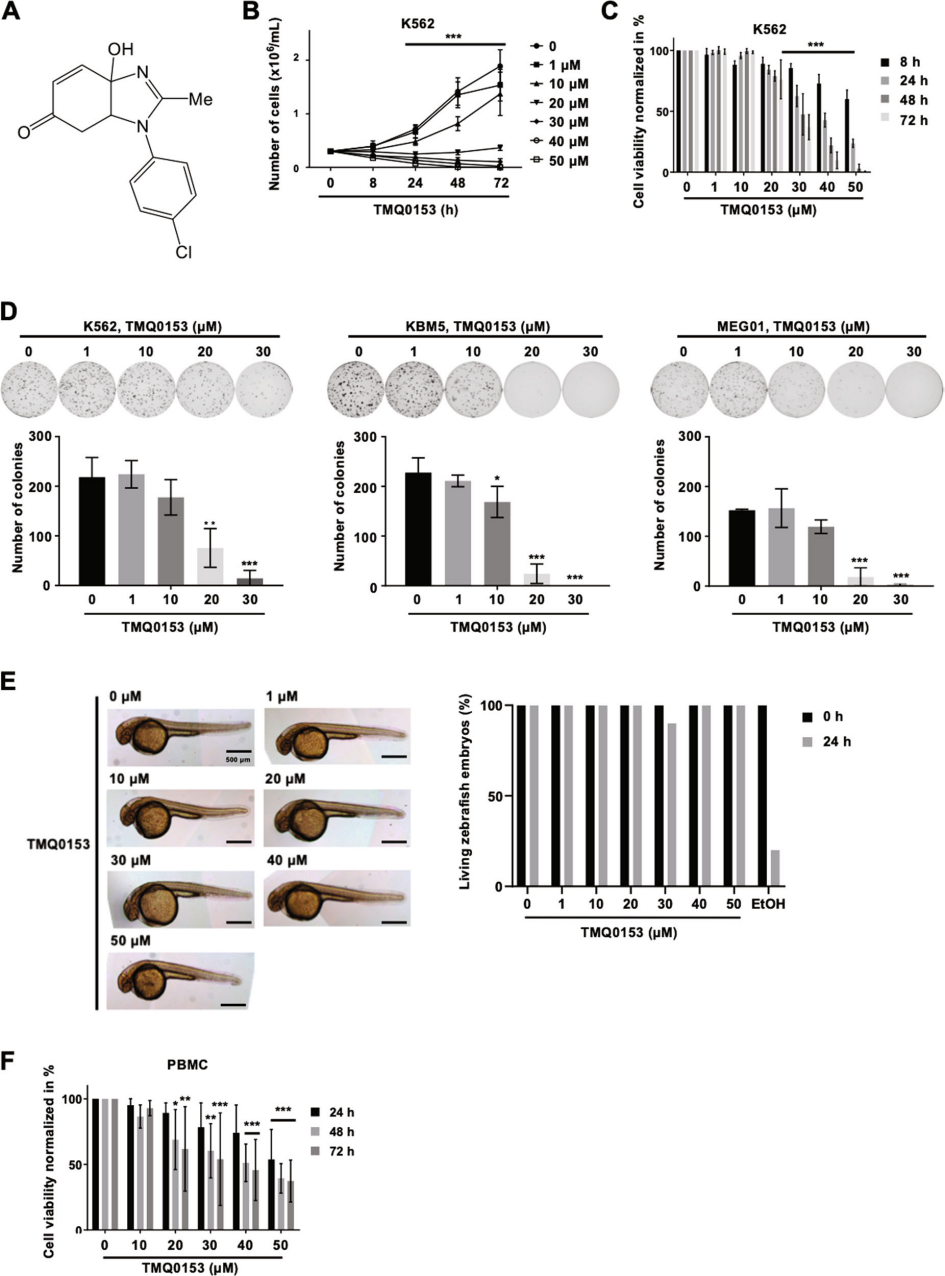
Effect of TMQ0153 on chronic myeloid leukemia cell viability. **a** Chemical structure of TMQ0153. **b** Time- and **c** dose-dependent effect of TMQ0153 on K562 cell proliferation (left panel) and viability (right panel). **d** Inhibitory effect of increasing concentrations of TMQ0153 on the colony forming capacities of the indicated CML cell lines. Upper panel: pictures representative of three independent experiments. Lower panel: quantification of the colony numbers. **a–d** All data represent mean (±S.D.) of three independent experiments. **e** Acute toxicity assay on zebrafish embryos after 24 h of treatment with increasing concentrations of TMQ0153. Pictures are representative of 10 fishes used for each condition (left panel) and the corresponding quantification of viable embryos (right panel). Ethanol (EtOH, 70%) was used as a positive control for toxicity. **f** Cytotoxicity of TMQ0153 on human peripheral blood mononuclear cells (PBMCs) by Trypan blue assay after 24, 48, and 72 h of treatment (PBMC data represent the mean (±S.D.) of five independent experiments). Statistical significance was assessed as *p < 0.05, **p < 0.01, ***p < 0.001 compared to untreated cells. Two-way ANOVA (Cell viability); post hoc: Sidak’s test. One-way ANOVA (Colony formation); post hoc: Sidak’s test. Two-way ANOVA (PBMC toxicity); post hoc: Dunnett’s test.

We then validated the effect of TMQ0153 (Fig. 1a) on imatinib-sensitive (Fig. 1b, c) and -resistant K562 (K562R), KBM5, imatinib-resistant KBM5 (KBM5R) and MEG01 cells (Supplementary Fig. 2A–D). We also compared the capacity of imatinib to induce cell death in K562 and K562R (Supplementary Fig. 3). Moreover, TMQ0153 decreased the colony formation capacity of various imatinib-sensitive and resistant CML cell types (Fig. 1d and Supplementary Fig. 2E). To extend our findings to an in vivo situation, zebrafish K562 xenograft formation was dose-dependently reduced by TMQ153, compared to controls (Supplementary Fig. 4). To assess for acute toxic side effects, no morphological alterations or toxicity of developing zebrafish larvae were observed at concentrations up to 50 µM, confirming the safety of this compound (Fig. 1e). We generalized our findings by using solid tumor cell lines lung A549, prostate PC3 and breast MCF7 cancer cells where TMQ153 also induced cell death (Supplementary Table II). Next, we evaluated the selectivity of TMQ0153. We conducted viability assays on peripheral blood mononuclear cells (PBMCs) isolated from healthy donors (Fig. 1f). Viability of TMQ0153-treated PBMCs was dose-dependently impacted. We then evaluated the differential toxicity. After 72 h of treatment, TMQ0153 at 40 and 50 µM was 6.8 and 149-fold more selective to K562 cells compared to PBMCs, respectively (Fig. 1f; Supplementary Table III). Considering the anti-cancer potential and differential toxicity, we selected CML models to further elucidate the mechanisms of action of TMQ0153.

### TMQ0153 induces concentration-dependent differential cell death modalities

**Fig. 2:**
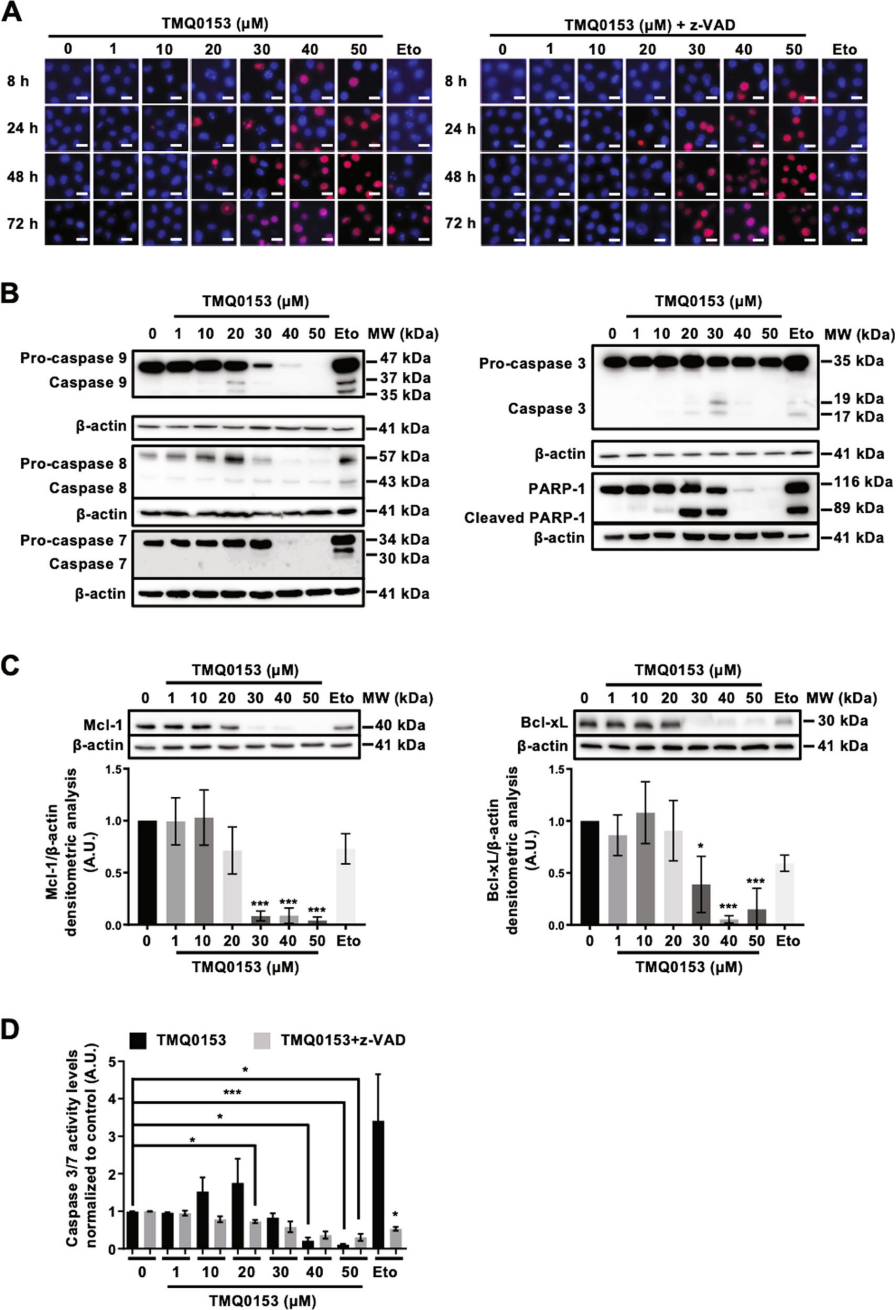
TMQ0153 triggered a concentration-dependent induction of caspase-dependent and independent non-apoptotic cell death in K562 cells. **a–d** K562 cells were treated with various concentrations of TMQ0153 in presence or absence of the pan caspase inhibitor carbobenzoxy-valyl-alanyl-aspartyl-[O-methyl]-fluoromethylketone (z-VAD; 50 μM). **a** After 8, 24, 48, and 72 h of treatment the type of cell death triggered by TMQ0153 was characterized by fluorescence microscopy after Hoechst/propidium iodide (PI) staining. Pictures representative of three independent experiments (top panel). Etoposide (Eto; 100 µM, 24 h) was used as a positive control for apoptosis induction. Scale bar: 25 µM. **b** Analysis of caspase and poly [ADP-ribose] polymerase (PARP)-1 cleavage by western blot after 24 h of treatment. **c** Effect of 24 h of treatment on Mcl-1 and Bcl-xL protein expression levels (top panels) and the corresponding densitometric analysis (middle and lower panels). **b**, **c** β–actin was used as loading control. **d** Quantification of caspase-3/7 activity levels after 24 h of treatment. Etoposide (Eto; 100 µM, 24 h) was used as a positive control for apoptosis induction. All pictures are representative of three independent experiments and graphs represent the mean (±S.D.) of three independent experiments. Statistical significance was assessed as *p < 0.05, **p < 0.01, ***p < 0.001 compared to untreated cells. Two-way ANOVA (microscopy analysis); post hoc: Sidak’s test. One-way ANOVA (caspase-3/7 assay); post hoc: Tukey’s test. One-way ANOVA (western blot quantification); post hoc: Dunnett’s test.

We then investigated the cell death mechanism induced by TMQ0153. Altogether, as shown in Fig. 2a and Supplementary Figs 5A and D, apoptotic cell death was induced at concentrations up to 20 μM after 24 h, rescued by z-VAD pretreatment. Non-apoptotic, z-VAD-insensitive cell death was dose-dependently induced at concentrations over 30 μM after 8, 24, 48, and 72 h (Fig. 2a and Supplementary Fig. 5C–H) confirming a concentration-dependent induction of caspase-dependent and -independent cell death modalities in both K562 and K562R cells. As shown in Fig. 2b, after 24 h of treatment with TMQ0153, procaspase-9, -8 and -3 were significantly activated leading to PARP-1 cleavage. TMQ0153 treatment led also to a decrease of the anti-apoptotic proteins myeloid cell leukemia (Mcl)−1 and B-cell lymphoma-extra large (Bcl-xL) in a dose-dependent manner (Fig. 2c). We confirmed our findings by measuring an increased caspase 3/7 activity following a treatment with up to 20 µM TMQ0153 for 24 h, which was abrogated in the presence of z-VAD (Fig. 2d).

### TMQ0153 activates necrostatin-1 sensitive necroptotic cell death

**Fig. 3:**
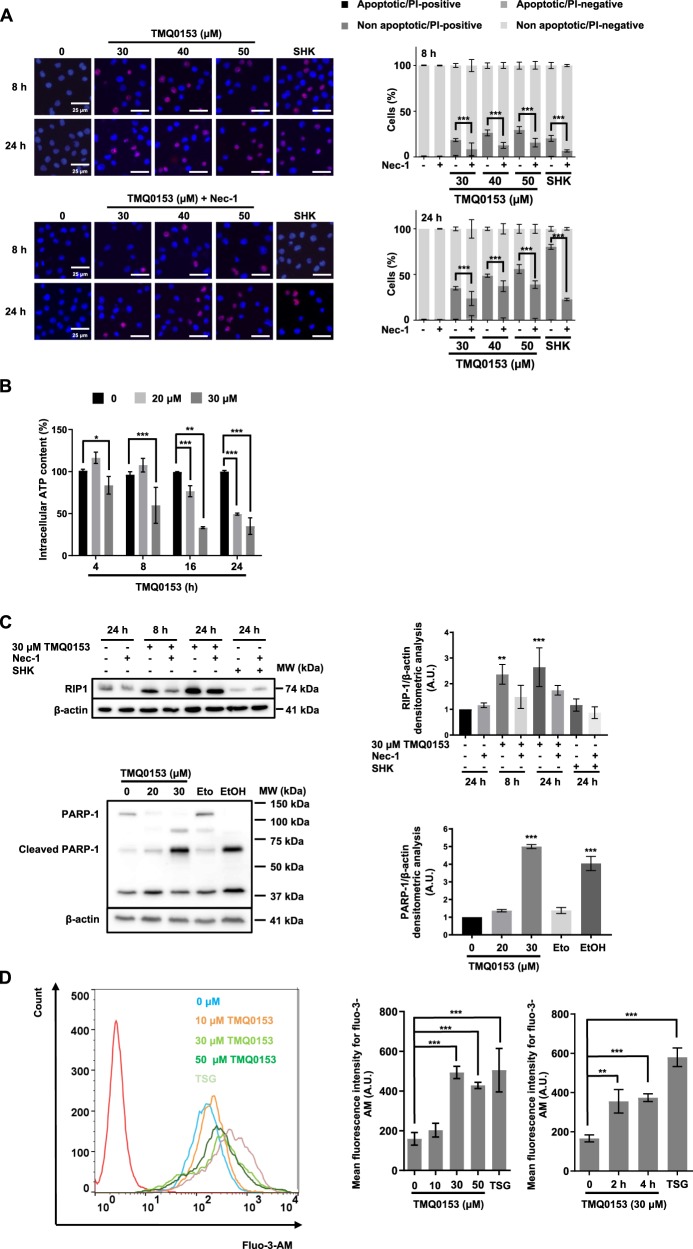
TMQ0153 induced a necrostatin-1-sensitive type of cell death in K562 cells. **a** K562 cells were incubated in presence or absence of 60 µM necrostatin (Nec)-1 for 1 h before a treatment with the indicated concentrations of TMQ0153. Shikonin (SHK; 5 µM) was used as a positive control for necrosis induction. **a** Nuclear morphology analyses by fluorescence microscopy following Hoechst/ propidium iodide (PI) staining after 8 and 24 h of treatment. Pictures representative of three independent experiments (left panels) and the corresponding quantification (right panels). **b** Measurement of intracellular ATP levels at the indicated concentrations and time points. **c** Receptor-interacting protein kinase (RIP)1 protein level and PARP-1 cleavage (left panel) were determined by western blotting using C2-10 antibody and the corresponding densitometric analysis (right panel). Shikonin (SHK; 5 µM, 24 h) and necrostatin (Nec)−1 (60 µM, 1 h) were used as a positive control and inhibitor for RIP1. Etoposide (Eto; 100 µM, 24 h) and ethanol (EtOH; 10 %, 2 h) were used as positive controls for apoptotic and necrotic PARP-1 cleavage, respectively. **d** Cytosolic Ca2^+^ levels were measured using Fluo-3-AM after 24 h (left panel) and time-dependently measured Ca^2+^ levels at 30 μM (right panel). Thapsigargin (TSG; 300 nM, 24 h) was used as a positive control for intracellular Ca^2+^ accumulation. β–actin was used as loading control. All pictures are representative of three independent experiments and all graphs represent the mean (±S.D.) of three independent experiments. Statistical significance was assessed as *p < 0.05, **p < 0.01, ***p < 0.001 for the indicated comparisons. Two-way ANOVA (microscopy analysis, cell viability); post hoc: Sidak’s test. Two-way ANOVA (Cell Titer Glo assay); post hoc; Tukey’s test. One-way ANOVA (intracellular Ca^2+^ assay); post hoc; Dunnett’s test. One-way ANOVA (western blot quantification); post hoc; Dunnett’s test.

We then investigated the mechanisms involved in TMQ0153-mediated non-apoptotic cell death at higher concentrations (i.e., from 30 µM) after 8 and 24 h in K562 cells. We pre-treated K562 cells with the RIP1 inhibitor Nec-1 to assess for necroptosis induction. Briefly, we confirmed that Nec-1 had no effect on apoptosis at lower concentrations after 24 h in K562 cells (i.e., up to 20 µM) by TMQ0153 treatment. Results are shown in Supplementary Fig. 6. TMQ0153 induced over 50% of non-apoptotic, PI positive cells after 24 h of treatment with 50 µM. Nec-1 pretreatment significantly prevented non-apoptotic cell death induction between 30–50 µM after 24 h, compared to shikonin, used as a positive control (Fig. 3a). These results indicated that TMQ0153 induced cell death in K562 cells via the necroptotic cell death pathway.

Necroptosis is known to be accompanied by a modulation of intracellular ATP, increased expression levels of RIP1 and the appearance of a non-apoptotic cleavage fragment of PARP-1 between 50 and 75 kDa^27^. Our results showed a significant decrease in intracellular ATP levels after 4, 8, 16 and 24 h at 30 µM TMQ0153 compared to 20 µM (Fig. 3b). Western blot results confirmed that TMQ0153-treated cells presented increased RIP1 expression levels and this expression was inhibited by a Nec-1 pretreatment. Also, the differentially cleaved necrotic PARP1 fragment revealed by the C2-10 antibody, supported that TMQ0153 induced necrotic cell death at 30 µM after 24 h of treatment (Fig. 3c). Besides, we observed a concomitant accumulation of cytosolic Ca^2+^ levels at 30 and 50 µM TMQ0153 after 24 h (Fig. 3d). Hematopoietic cell lines largely lack RIP3 expression and this loss of RIP3 may reduce sensitivity against cytotoxic agents^28^.

We then investigated the basal expression levels of RIP3 by western blot analysis in different cancer cell types with known RIP3 expression levels. As expected, RIP3 was silenced in K562, A549, PC3 and MCF7 cells (Supplementary Fig. 7A). We then treated K562 cells with the DNA hypomethylating agent 5-aza-2’-deoxycytidine (5-aza) at 1 µM for 6 days to re-express RIP3. As shown in Supplementary Figs 7B, 5-aza treatments increased RIP3 protein expression levels. We then co-treated these cells with TMQ0153 to investigate potential sensitization. Results showed that TMQ0153 treatment of 5-aza-pretreated K562 cells led to significantly enhanced cell death levels (54%) compared to TMQ0153 treatment alone (21%). Overall, our results indicated that the cytotoxicity of TMQ0153 in K562 cells is mediated by necroptosis at higher concentrations. In addition, a pretreatment with DNA hypomethylating agents like 5-aza may further potentialize the effect of TMQ0153 in CML cells devoid of RIP3.

### TMQ0153 induces an early onset of autophagy in K562 cells followed by controlled necrosis

**Fig. 4:**
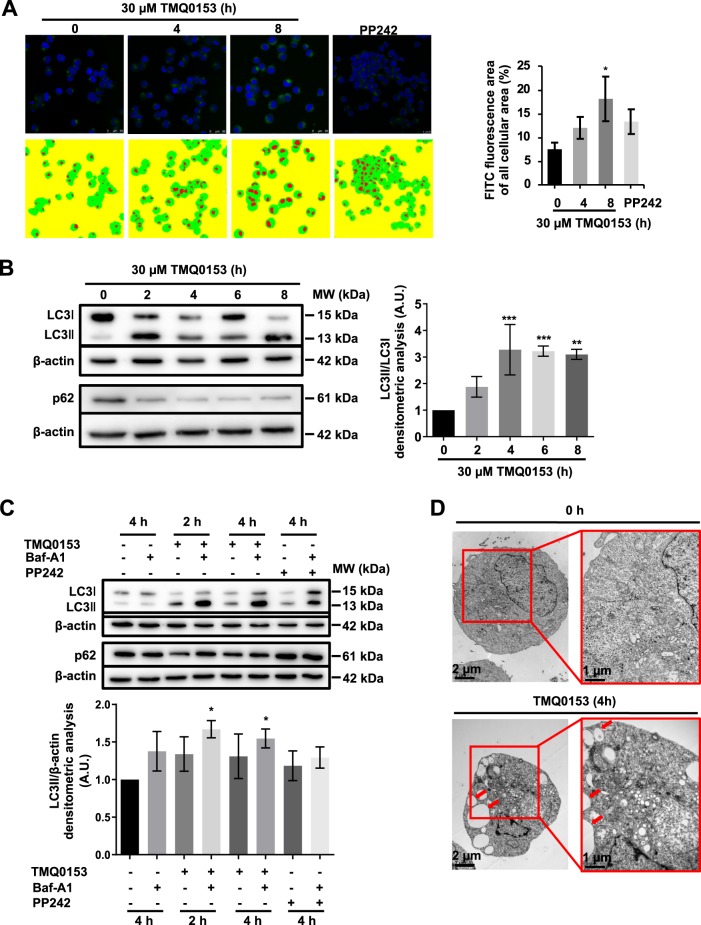
TMQ0153 stimulated autophagy prior to necroptosis. **a** Confocal UV microscopy analysis after staining with Cyto-ID (left panel). Representative images of FITC stained images in the 4 groups accompanied by the corresponding pseudocolor masks (red: FITC signal, green: cell area) used for calculation and the corresponding quantification of fluorescence intensity (right panel). Statistical results were compared by Kruskal–Wallis test followed by Conover post-test further adjusted by the Benjamini-Hochberg FDR method. 0 h vs. 4 h and vs 8 h (*p* < 0.0004 and *p* < 0.00006, respectively), 4 h vs.8 h (*p* < 0.002). **b** Western blot detection of LC3 and p62 protein levels (left panel) and the corresponding densitometric analysis (right panel). **c** Similar analysis in cells pretreated with 40 nM bafilomycin A1 (baf-A1) for 1 h (upper panel) and the corresponding densitometric analysis (lower panel). 10 µM PP242 for 4 h was used as a positive control for autophagy induction. **d** Transmission electron microscopy at ×12.000 and ×25.000 magnification: arrows indicate autophagolysosomes. Statistical significance was assessed as *p < 0.05, **p < 0.01, ***p < 0.001 compared to untreated cells unless otherwise specified. One-way ANOVA (western blot quantification); post hoc; Tukey’s test. Two-way ANOVA (mito stress test); post hoc; Sidak’s test.

As we observed extensive vacuole formation (Supplementary Fig. 8A) in treated K562 cells prior to cell death induction, we hypothesized that TMQ0153 was triggering an initial intracellular stress reaction, potentially via autophagy. TMQ0153 dose-dependently and progressively induced the conversion of LC3-I to LC3-II between 10 and 50 µM after 2 h by western blot analysis. Results showed that TMQ0153 significantly increased LC3-II levels at 10 µM (Supplementary Fig. 8B). In addition, there is a significant increase of LC3 conversion between 10 and 30 µM potentially reflecting the differential ATP levels in the treated cells. Based on these results, an initial morphological analysis of TMQ0153-treated K562 cells by confocal microscopy and Diff-Quik staining allowed quantifying early vesicle formation (Supplementary Fig. 8C). Treatment of K562 cells with TMQ0153 at necroptosis-inducing concentrations (30 µM) for 0, 4, 8 h compared to PP242 showed the onset of vesicle formation by confocal microscopy after CYTO-ID staining, further ascertaining autophagic activity (Fig. 4a). Western blot analysis demonstrated that TMQ0153 time-dependently induced the conversion of LC3-I to LC3-II at 30 µM after 2, 4, 6, and 8 h, prior to necroptosis induction. In agreement with these results, sequestosome-1 (SQST)M1/p62 (Fig. 4b) was degraded. Moreover, in the presence of the lysosome inhibitor baf-A1, we observed an enhanced accumulation of LC3-II and p62 levels in TMQ0153-treated cells after 2 and 4 h confirming an active autophagic flux (Fig. 4c). Results were confirmed by transmission electron microscopy (TEM) confirming extensive vacuolization (Fig. 4d).

**Fig. 5:**
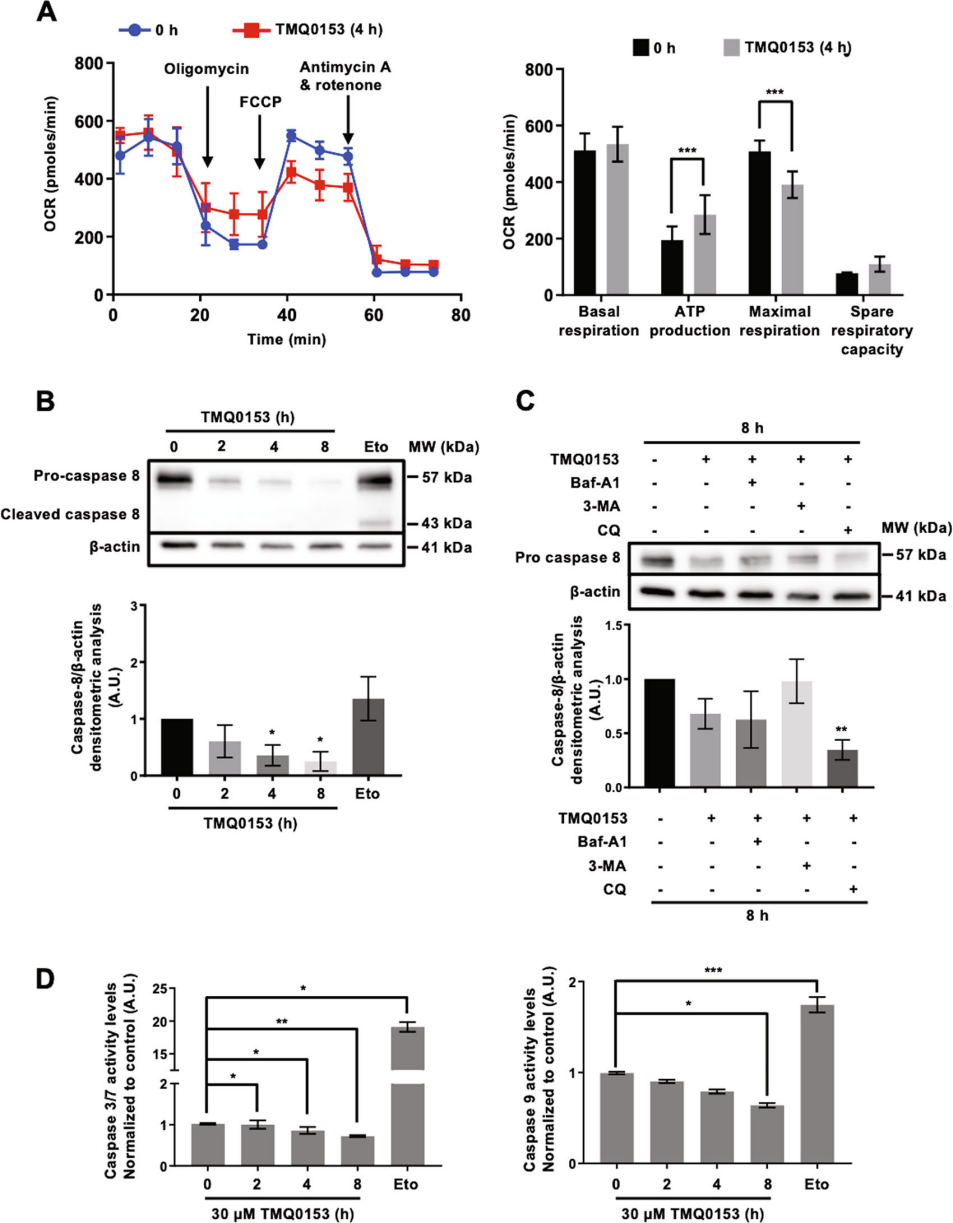
Effect of TMQ0153 on cellular metabolism. **a** Oxygen consumption rate (OCR) was measured by Seahorse XFp analyzer. **b** Caspase 8 analysis by western blot (upper panel) and the corresponding densitometric analysis (lower panel). Etoposide (Eto; 100 µM, 24 h) was used as a positive control for apoptotic caspase cleavage. **c** Caspase 8 analysis by western blot (upper panel) in the presence or absence of autophagy inhibitors: 40 nM baf-A1, 10 mM, 3-methyladenine (3-MA) and 75 μM chloroquine (CQ) and the corresponding densitometric analysis (lower panel). In western blot analyses, β-actin was used as a loading control. **d** Quantification of caspases-3/7 (left graph) and -9 activity (right graph) levels at 30 µM of TMQ0153 treatment. Etoposide (Eto; 100 µM, 24 h) was used as a positive control for apoptosis induction. All pictures are representative of three independent experiments and data represent mean (±S.D.) of three independent experiments. Statistical significance was assessed as *p < 0.05, **p < 0.01, ***p < 0.001 compared to untreated cells unless otherwise specified. One-way ANOVA (Caspase-3/7 and -9 assay); post hoc; Tukey’s test. Two-way ANOVA (mito stress test); post hoc; Sidak’s test. One-way ANOVA (western blot quantification); post hoc; Dunnett’s test.

To investigate the effect of TMQ0153 on cellular metabolism, we used a Seahorse XF Analyzer. Our results showed a significant decrease in the oxygen consumption rate (OCR) upon 4 h of treatment with 30 µM TMQ0153 (Fig. 5a), which precedes necroptosis induction. As these results indicated that TMQ0153 disrupted mitochondrial bioenergetics, we further detected mitochondria with damaged morphologies by TEM (4 h, 30 µM) in line with the observed metabolic alterations.

We then investigated the effect of autophagy induction on caspase-8 expression levels. Our results showed a progressive reduction of pro-caspase-8 levels during autophagy induction between 2 and 8 h with 30 µM TMQ0153 (Fig. 5b). Despite the progressive degradation of pro-caspase-8 after treatment, Fig. 5c shows that chemical inhibition of autophagy at 8 h by Baf-A1, 3-MA and CQ does not allow to significantly rescue pro-caspase-8 levels. Noteworthy, under the same conditions, caspase-3/7 and -9 activities significantly decreased (Fig. 5d).

### Autophagic inhibitors augment necroptosis induced by TMQ0153 in K562 cells

**Fig. 6:**
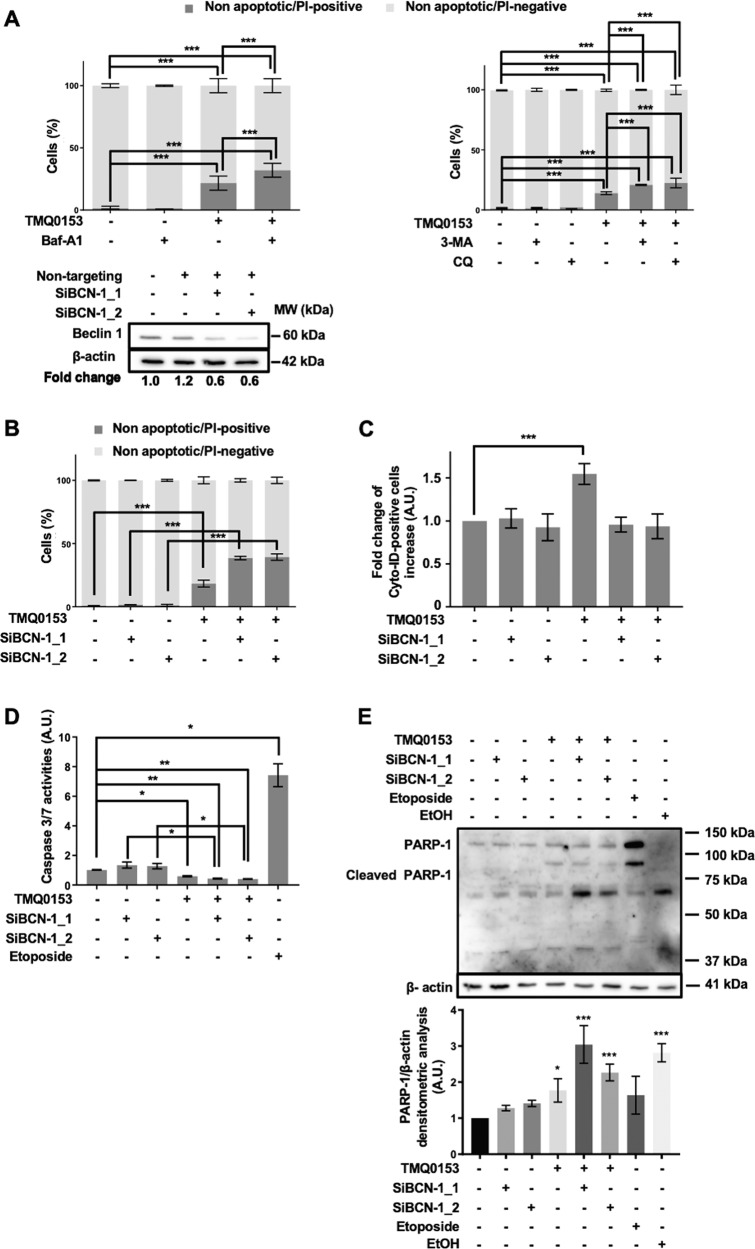
Inhibition of autophagy increases TMQ0153-induced necroptosis. **a** Effect of autophagy inhibitors (40 nM baf-A1, 10 mM, 3-methyladenine (3-MA) and 75 μM chloroquine (CQ)) on death of K562 cells treated with 30 µM of TMQ0153 assessed by nuclear morphology analysis after 8 h of treatment. **b-e** K562 cells were transfected with specific small interfering (si)RNAs against beclin 1 [(SiBCN-1), 5 nM SiBCN-1_1 and 10 nM SiBCN-1_2] for 24 h. **b** Upper panel: effect of siRNA on beclin 1 protein expression level. After quantification of the bands, beclin 1 levels were normalized to β-actin. Lower panel: transfected cells were treated with 30 μM of TMQ0153 and a nuclear morphology analysis was carried out after 8 h of treatment. **c-e** Effect of siRNA on TMQ0153-induced autophagy quantified by flow cytometry after Cyto-ID staining (**c**); on caspase-3/7 activity (**d**), and PARP-1 cleavage using C2-10 antibody (**e**). Etoposide (Eto; 100 µM, 24 h) and EtOH (10 %, 2 h) were used as positive controls for apoptotic and necrotic PARP-1 cleavage (upper panel) and the corresponding densitometric analysis of necrotic cleavage (lower panel) is visualized. In western blot analyses, β-actin was used as a loading control. All pictures are representative of three independent experiments and data represent mean (±S.D.) of three independent experiments Statistical significance was assessed as *p < 0.05, **p < 0.01, ***p < 0.001 for the indicated comparisons. Two-way ANOVA (nuclear morphology analysis); post hoc; Sidak’s test. One-way ANOVA (Cyto-ID assay); post hoc; Dunnett’s test. One-way ANOVA (caspase 3/7 assay); post hoc; Tukey’s test. One-way ANOVA (western blot quantification); post hoc; Dunnett’s test.

To investigate the relationship between TMQ0153 induced autophagy and necroptosis, K562 cells were treated with TMQ0153 in the presence of autophagic inhibitors. Nuclear morphology analyses showed that the inhibition of TMQ0153-induced autophagy by baf-A1 significantly enhanced necroptosis in K562 cells compared to cells treated with TMQ0153 alone (Fig. 6a). In addition, baf-A1-, 3-MA- and CQ-mediated inhibition of autophagy led to necroptotic cell death in line with a switch from apoptotic to necrotic cell demise and concomitant with the reduction of procaspase-8 levels (Fig. 5b).

To avoid non-specific effects of chemical inhibitors, we then also investigated the effect of TMQ0153-induced autophagy in beclin 1-siRNA-transfected cells. Silencing beclin 1 enhanced necroptosis (Fig. 6b) and decreased autophagy (Fig. 6c). Moreover, enhanced levels of necrotic PARP-1 cleavage products (50–75 kD) were detected when using the C2-10 anti-PARP antibody (Fig. 6d, e). Altogether, whereas inhibition of autophagy did not allow a significant switch towards apoptosis, it caused increased levels of necrosis, most likely due to induction of energetic catastrophe.

### CML patient cells are characterized by increased expression levels of genes involved in oxidative stress

We wanted to further document the status of activated redox metabolism by using patient data. Further exacerbation of this status should facilitate cell death induction in agreement with the concept of pro-oxidant CML therapy. Indeed. CML patient cells are characterized by increased expression levels of genes involved in oxidative stress. We used a patient cohort regrouping 74 healthy donors and leukemia patients including 76 patients that were diagnosed with CML^22,23^ to assess the gene expression level of oxidative stress-related genes. In particular, we were interested in assessing the gene expression level of the NADPH oxidases (NOX) family constituted of 7 members (NOX1-NOX5, DUOX1, DUOX2), as they are largely responsible for the production of ROS^29^. Our results show that the expression level of NOX regulator cytochrome b-245 heavy chain (CYBB) was more elevated in CML patients compared to healthy donors (Supplementary Fig. 9) leading to an increased NOX activity and thus an increase in ROS production^30^. Based on these observations, we hypothesized that the resulting increased ROS levels in CML patient cells could be therapeutically targeted by exacerbation of intracellular ROS levels by TMQ0153 leading to a pro-oxidant treatment approach. Indeed, from a chemical point of view, TMQ0153 can be redox active similar to quinones and afford radical anions leading to the formation of reactive superoxide.

### TMQ0153 depolarizes mitochondrial membrane potential and triggers necroptotic cell death through ROS formation

**Fig. 7:**
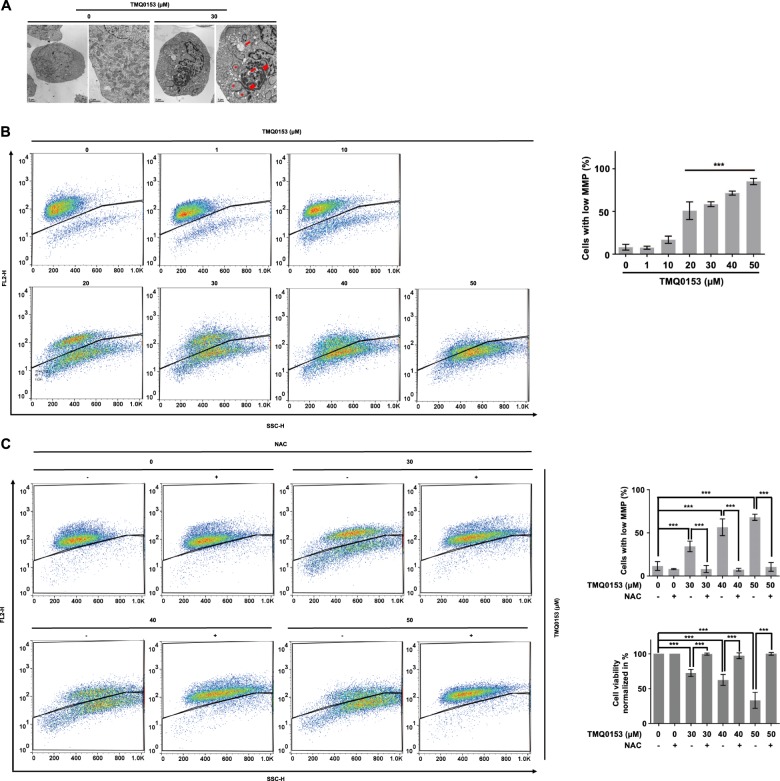
TMQ0153 induced mitochondrial dysfunction and MMP in K562 cells. **a** Cells were treated with TMQ0153 for 8 h and mitochondrial morphology was assessed by TEM at 12.000x and 25.000x magnification. Single arrows and double arrows indicate respectively dilated and giant mitochondria, asterisks indicate autophagophores. **b** Mitochondrial membrane potential (MMP) analysis in cells treated with increasing concentrations of TMQ0153 for 24 h. The fraction of low MMP presenting cells is depicted. **c** Cells were pre-incubated for 1 h in presence or absence of 50 mM N-acetyl-L-cysteine (NAC) followed by a treatment with the indicated concentrations of TMQ0153. After 24 h of treatment, MMP (upper panel) cell viability (bottom panel) were assessed by trypan blue assay and flow cytometry, respectively. All pictures are representative of three independent experiments and data represent the mean (±S.D.) of three independent experiments. Statistical significance was assessed as *p < 0.05, **p < 0.01, ***p < 0.001 compared to untreated cells unless otherwise specified. One-way ANOVA (mitochondrial membrane); post hoc; Dunnett’s test. One-way ANOVA (cell viability); post hoc; Tukey’s test.

**Fig. 8:**
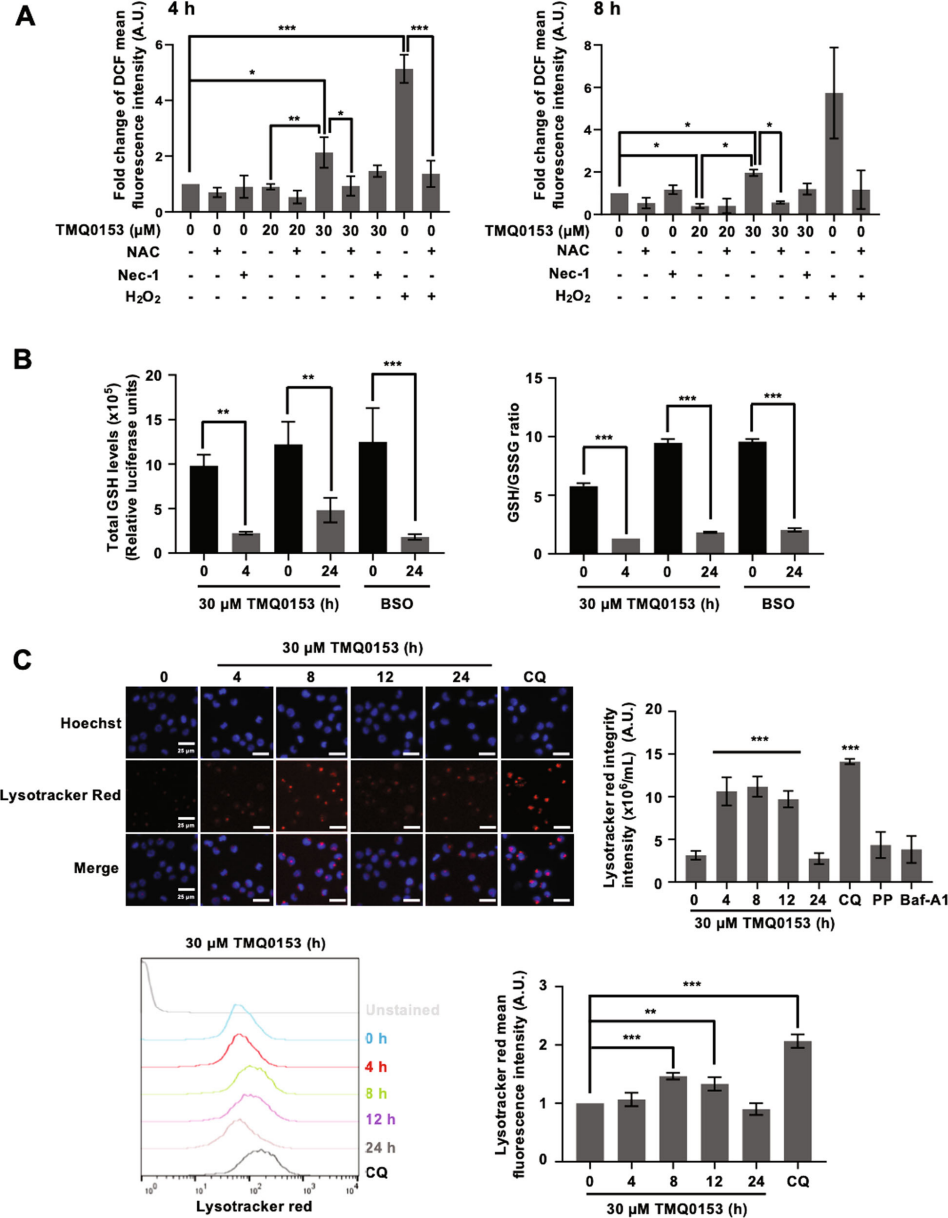
TMQ0153 triggers necroptotic cell death through decreased GSH levels, involvement of LMP and ROS formation. **a** Cells were pre-incubated for 1 h in presence or absence of 50 mM NAC or 60 µM necrostatin (Nec)−1. After 4 and 8 h of treatment with TMQ0153 at 20 and 30 µM, reactive oxygen species (ROS) levels were measured by flow cytometry following dichlorofluorescein diacetate (H_2_DCFDA) staining. H_2_O_2_ was used as a positive control for ROS induction. **b** Quantification of total GSH levels (left panel) and GSH (glutathione)/glutathione disulfide (GSSG) ratio (right panel). 50 µM Buthionine sulfoximine (BSO) was used as a positive control for the inhibition of GSH synthesis. **c** Cells were stained with Hoechst and Lysotracker Red and analyzed by fluorescence microscopy. Lysotracker Red fluorescence intensity was quantified using Image J 1.8.0 software (upper panel). Lysotracker Red intensity was quantified by FACS (bottom panel). Chloroquine (CQ; 75 μM, 4 h), PP242 (PP, 10 μM, 4 h) and baf-A1 (40 nM, 4 h) were used as a positive and negative controls for autophagy inhibition and induction, respectively. All pictures are representative of three independent experiments and data represent the mean (±S.D.) of three independent experiments. Statistical significance was assessed as *p < 0.05, **p < 0.01, ***p < 0.001 compared to untreated cells unless otherwise specified. One-way ANOVA (LMP); post hoc; Dunnett’s test. One-way ANOVA (ROS, GSH assay); post hoc; Tukey’s test.

Accumulation of intracellular ROS is known to depolarize the mitochondrial membrane potential (MMP). First, we performed a morphological analysis of mitochondria by TEM after treatment of K562 with TMQ0153 (30 µM). Our results showed that 30 µM TMQ0153 induced mitochondrial morphological changes such as enlarged and swollen mitochondria compared to control after 8 h (Fig. 7a and Supplementary Fig. 10A, B). Next, we measured the MMP in K562 cells after 24 h of treatment with TMQ0153. Results demonstrated that TMQ0153 increased the proportion of cells with low MMP in a dose-dependent manner (Fig. 7b). In addition, TMQ0153-mediated reduced cell viability and MMP loss were prevented by the ROS scavenger NAC (Fig. 7c) and also prevented by the RIP1 inhibitor Nec-1 (Supplementary Fig. 10C). Next, we investigated the implication of ROS in the generation of necroptotic cell death^31^. To identify the nature and roles of intracellular ROS induced by TMQ0153, K562 cells were pretreated with and without antioxidants NAC, Trolox and Tiron. Apoptotic concentrations of TMQ0153 (20 µM) did not induce significant levels of ROS compared to a necrosis-inducing concentration (30 µM) after 4 and 8 h whereas pretreatment with NAC significantly abrogated ROS formation at 30 µM (Fig. 8a and Supplementary Fig. 11A) in both K562 and K562R cells. Vitamin E derivative Trolox that reduces the levels of lipid peroxidation when the oxidation was initiated inside the plasma membrane^32^ or mitochondrially-localized Tiron^33^ did not significantly reduce ROS levels (Supplementary Fig. 11B).

Since the balance of intracellular reduced and oxidized glutathione (GSH) levels reflects the redox state of the cells, we evaluated total GSH levels and the GSH/GSSG ratio after 4 and 24 h of treatment. Results revealed that both total GSH levels and GSH/GSSG ratio were significantly reduced upon 30 µM TMQ0153 treatment (Fig. 8b). After GSH depletion, redox and cellular stress are considered to be potent inducers of mitochondrial dysfunction through ROS accumulation^34^. Therefore, we examined the effect of Nec-1 on intracellular ROS accumulation by TMQ0153 and found that Nec-1 pretreatment decreased cellular ROS levels in TMQ0153-treated K562 cells after 4 and 8 h (Fig. 8a). This finding could suggest that Nec-1 protects against TMQ0153-induced necroptosis by suppressing the intracellular burden of ROS formation after necroptosis induction. Finally, ROS-induced lysosomal membrane potential (LMP) is increasing in K562 cells after TMQ0153-induced necroptotic cell death in a time-dependent manner at 30 µM as demonstrated by fluorescence microscopy and FACS (Fig. 8c).

### TMQ0153-treated K562 cells release immunogenic cell death markers

Our results showed a decrease in intracellular ATP levels and increased mitochondrial ROS and cytoplasmic Ca^2+^ levels eventually concomitant with necroptosis induction^35^. Since these changes were described to contribute to the immunogenicity of the dying cells^36^, we also assessed the release of HMGB1 and demonstrated that TMQ0153 triggered accumulation HMGB1 in the supernatant in a dose- and time-dependent manner in K562 cells (Supplementary Fig. 12A). In addition, 24 h of treatment with TMQ0153 induced extracellular ATP secretion into the supernatant of K562 cells at 30 and 50 µM (Supplementary Fig. 12B). Furthermore, the potential immunogenic signal from dying cells also includes proteins that are exposed at the surface of stressed or dying cells. Results showed that TMQ0153-treated cells increased significantly the ectopic expression of calreticulin (Ecto-CRT) (Supplementary Fig. 12C) and Ecto-ERp57 (Supplementary Fig. 12D) by fluorescent microscopy and FACS. These results indicate that TMQ0153-induced necroptosis could further augment immunogenicity of dying K562 cells.

## Discussion

CML still has high morbidity and mortality among the leukemia patients^37^. Even though the development of TKIs is an effective treatment against CML, severe side effects and mutations of BCR-ABL are considered as one of main reasons for drug resistance^38^. For this reason, novel therapies that target the molecular or metabolic characteristics of CML are highly required.

In the present study, we attempted to address the interplay between apoptosis, autophagy, and necroptosis in CML cell models, by using an experimental pro-oxidant therapeutic approach with the cytotoxic synthetic hydroquinone derivative TMQ0153 aiming to disrupt oxidative and metabolic stress homeostasis. As a result, we observed a ROS- and concentration-dependent induction of protective autophagy eventually leading to Nec-1-sensitive necroptosis, whereas low TMQ0153 concentrations do not trigger any significant increase in ROS levels and led to caspase-dependent apoptosis. We also observed different levels of ATP during the induction of apoptosis and necroptosis. Indeed, ATP is required for caspase activation and induction of apoptosis^39^. Therefore, high levels of ROS can lead to necroptotic cell death whereas low levels of ROS induce apoptotic cell death.

Quinone derivatives such as 5-hydroxy-2-methyl-1,4-naphthoquinone (plumbagin) induce apoptosis or necrosis in AML by producing ROS and decreasing Mcl-1 and Bcl-2 anti-apoptotic protein abundance^40^. However, the effect of quinone-induced ROS on the apoptotic machinery remains largely unknown. In our study, 20 µM TMQ0153 induced apoptosis in K562 cells along with caspase 8, 9 and 3 cleavage, that are both prevented in the presence of z-VAD. In addition, we found that apoptosis was associated with an early downregulation of anti-apoptotic protein Mcl-1 and Bcl-xL after TMQ0153 treatment.

We showed that TMQ0153 induces cell death in K562 cells *via* a RIP1-dependent necroptotic cell death pathway. 2,3,5-tris-hydroquinone was reported to induce ROS production and increase intracellular Ca^2+^ levels that contribute to PARP-1-mediated necrosis in HK-2 cells^41^. β-lapachone is reduced to β-lapachone hydroquinone, which induces programmed necrosis through the ROS production and a RIP1-dependent cell death pathway in human hepatocellular carcinoma^42^. Here, TMQ0153 is acting as a pro-oxidant and induces necroptosis through downstream mediators including RIP1 leading to mitochondrial dysfunction as a response to energy depletion. Previous research investigated that cells lacking RIP3 expression were resistant to typical programmed necrotic stimuli but became sensitive when RIP3 is re-expressed^28^. Our results supported these results as we observed a sensitization against TMQ0153 by the re-expression of RIP3 after treatment with the DNA demethylating agent 5-azacitidine^28^.

Despite the successful use of TKIs in CML therapy, a better understanding of the physiology of CML cells remains important. According to Karvela et al.^43^, CML cells show elevated rates of a basal autophagic flux due to upregulation of autophagy genes including ATG7. In addition, higher levels of ROS and autophagy were shown to be implicated in the development of CML and closely involved in drug resistance in leukemia^44,45^. Our results confirmed the elevated basal levels of autophagosome formation in K562 cells. Autophagy is triggered as a pro-survival strategy in human cancer cells treated with mammalian target of rapamycin (mTOR) inhibitor rapamycin^46^, sarco/endoplasmic reticulum (ER) Ca^2+^-ATPase (SERCA) inhibitor stemphol^18^ or the naphthoquinone shikonin^47^. Indeed, the inhibition of shikonin-induced autophagy increased necroptosis as well as PARP-1-mediated cell death in A549^47^. Our results support the idea that autophagy protects against necroptosis in TMQ0153-treated K562 as we confirmed that autophagy was detected at early time points before the induction of necroptotic cell death with 30 µM TMQ0153 most likely due to an early cellular stress response in K562 cells.

In cancer cells, metabolic stress may arise from insufficient energy or oxygen supply. Autophagy can be induced as an alternative source of energy and metabolites^48^. Our results showed that K562 cells treated with autophagy inhibitors such as baf-A1 enhanced necroptotic cell death concomitantly with a non-apoptotic degradation of caspase-8, thus preventing canonical apoptosis induction. These results were confirmed by a profile of PARP-1 degradation typical of necrosis/necroptosis.

In beclin 1-silenced K562 cells, TMQ0153 induced the appearance of necrotic PARP-1 band and enhanced necroptotic cell population suggesting that autophagy serves a protective role. In addition, we observed that TMQ0153 induced autophagic responses in K562 cells as evidenced by LC3-positive autophagy like vacuoles, and the increased conversion of LC3-I to LC3-II. We also found that pretreatment with baf-A1, which blocks autophagosomal degradation, increased the formation of LC3-II in K562 cells after treatment with TMQ0153.

We provided evidence that TMQ0153 induced the dysfunction of mitochondria as shown by TEM and OCR analysis, as well as oxidative stress *via* increasing ROS levels and triggering accumulation of cytoplasmic Ca^2+^ levels. GSH is an important antioxidant in cellular metabolism and the reduction of GSH levels was described to induce autophagy, apoptotic or necrotic/necroptotic cell death^49,50^. We observed that intracellular GSH levels decreased during the onset of autophagy and necroptosis induced by TMQ0153. TMQ0153, as a pro-oxidant compound, amplifies ROS stress in K562 cells prior to necroptosis induction. Therefore, necroptotic cell death potentially further exacerbates ROS generation. The change of oxidative stress levels by TMQ0153 affects mitochondria homeostasis leading to different forms of cell death. A shift from apoptosis to necroptosis appears to be associated with decreased mitochondrial function and ATP production^51^. Other mechanisms may involve Ca^2+^ accumulation known to trigger necroptotic cell death^14^.

Weak release of lysosomal enzymes leads to apoptosis, whereas a massive release of lysosomal enzymes results in necrosis^52^. In our study, TMQ0153 induced increased ROS and LMP at early time points which are decreased later when necroptosis is induced. Interestingly, increased ROS levels are specifically reduced by the pre-treatment by NAC but not by Trolox and Tiron. These compounds possess different antioxidant capacities. NAC is a precursor of glutathione and can scavenge different types of ROS^53^. Trolox neutralizes lipid-derived radicals^54^ whereas Tiron is a metal chelator^55^. Considering our results, ROS induced by TMQ0153 which is preventable by NAC, must be generated in the mitochondria considering the specific antioxidant effects of NAC^56^.

Wiedmer et al. showed that autophagy is upregulated for the clearance of damaged lysosomes, leading to cell recovery, thus playing a pro-survival role^57^. Hence, lysosomal instability triggered by TMQ0153 contributes to final steps of necroptosis. Besides LMP, mitochondrial dysfunction is another executioner of necroptosis^31^. Our results showed that TMQ0153 treatment generated mitochondrial alterations and dysfunction associated with a loss of MMP.

Necroptosis triggers cell membrane rupture and the release of cellular cytoplasmic contents into the extracellular spaces such as HMGB1 and ATP^13^. TMQ0153 treatment significantly released HMGB1 and increased extracellular ATP levels. Immunogenic cell death (ICD) is a form of chemotherapy-induced tumor cell death, which is mediated by damage associated molecular patterns (DAMPs) that triggers effective antitumor immune responses^58^. ICD-inducers are able to mediate endoplasmic reticulum (ER) stress resulting in the cell surface presentation of calreticulin and ERp57^13,59^. Here, we showed that TMQ0153 treatment led to CRT and ERp57 surface expression. These results provided evidence that TMQ0153 released ICD markers as an essential feature of this chemotherapeutic compound.

In summary (Supplementary Fig. 13, based on^60^ with modifications), TMQ0153 possesses a potent pro-oxidant capacity against imatinib-sensitive and -resistant CML cells underlining the interest of TMQ0153 as an experimental pre-clinical therapeutic agent accompanied by release of ICD markers.

## Supplementary information

Supplementary figure legends

Supplementary tables

Supplementary figure 1

Supplementary figure 2

Supplementary figure 3

Supplementary figure 4

Supplementary figure 5

Supplementary figure 6

Supplementary figure 7

Supplementary figure 8

Supplementary figure 9

Supplementary figure 10

Supplementary figure 11

Supplementary figure 12

Supplementary figure 13
