# Supplementary material for: Tetrahydrobenzimidazole TMQ0153 triggers apoptosis, autophagy and necroptosis crosstalk in chronic myeloid leukemia
Source: Cell Death Dis. 2020 Feb 7;11(2):109. doi: 10.1038/s41419-020-2304-8 (PMC7007439; doi:10.1038/s41419-020-2304-8)
Supplement: Supplementary file 1 — Supplementary figure legends [file 41419_2020_2304_MOESM1_ESM.docx]

**Supplementary materials**

**Tetrahydrobenzimidazole TMQ0153 triggers apoptosis, autophagy and necroptosis crosstalk in chronic myeloid leukemia**

**Song et al.**

**Suppl. Fig. 1. TMQ0153 accumulates in K562 cells in a dose- and time**-**dependent manner.** (**A**) TMQ0153 showed a concentration- and time-dependent cellular uptake as quantiﬁed by flow cytometry using FL1, FL2 & FL3 channels with the strongest shift in the FL3 channel. Left panel: representative dot blots obtained with FL3 channel. Right panels: fluorescence intensity quantification on FL1, FL2 & FL3 channels represented as the mean (± S.D.) of three independent experiments. Statistical significance was assessed as *p < 0.05, **p < 0.01, ***p < 0.001 compared to untreated cells. (**B**) After 24 h of treatment, autofluorescent TMQ0153 was detected by confocal microscopy. Two-way ANOVA (FACS); post hoc; Sidak’s test.

**Suppl. Fig. 2. The validation of the resistance to imatinib induced cell death in K562 and K562R cells.** **(A)** A dose-dependent comparison of imatinib-sensitive and -resistant K562 cells after 24 h. After 24 h of treatment, the type of cell death triggered by TMQ0153 was characterized by flow cytometry (FACS) after Annexin V APC/ propidium iodide (PI) staining. Pictures representative of three independent experiments (top panel) and the corresponding quantification (lower panel) are shown. Statistical significance was assessed as *p < 0.05, **p < 0.01, ***p < 0.001 compared to untreated cells. Two-way ANOVA (FACS); post hoc: Sidak’s test.

**Suppl. Fig. 3. Cytotoxic and anti-proliferative effects of TMQ0153 on CML cells.** Time- and dose dependent effect of TMQ0153 on the proliferation (left panel) and viability (right panel) of K562R (**A**)**,** KBM5 (**B**), KBM5R (**C**) and MEG01 (**D**) cell lines. (**E**) Inhibitory effect of increasing concentrations of TMQ0153 on the colony forming capacities of K562R. Left panel: pictures representative of three independent experiments. Right panel: quantification of the colony numbers. Data represent the mean (±S.D.) of three independent experiments. Statistical significance was assessed as *p < 0.05, **p < 0.01, ***p < 0.001. Two-way ANOVA; post hoc; Sidak’s test (Viability and proliferation). One-way ANOVA post hoc; Sidak’s test (colony formation assay).

**Suppl. Fig. 4. Inhibition of *in vivo* tumor forming ability by TMQ0153 of K562 cells xenografted in zebrafish.** Pictures [bright ﬁeld (top), cell tracker CM-Dil dye-stained cells (middle), merge (bottom] of the 9 fishes used for each condition. PBS injection was used as a control for injection toxicity. The intensity of fluorescence was quantified and represented as the mean (± S.D.) of 9 fishes. Scale bar: 500 μm. Statistical significance was assessed as ***p < 0.001 by One-way ANOVA; post hoc: Dunnett’s test.

**Suppl. Fig. 5. Concentration-dependent induction of caspase-dependent and independent non-apoptotic cell death in K562 and K562R cells.** (**A**) K562 cells were treated with various concentrations of TMQ0153 in presence or absence of the pan caspase inhibitor carbobenzoxy-valyl-alanyl-aspartyl-[O-methyl]-fluoromethylketone (z-VAD; 50 μM). (**A**) After 8, 24, 48 and 72 h of treatment the type of cell death triggered by TMQ0153 was characterized by fluorescence microscopy after Hoechst/ propidium iodide (PI) staining. The corresponding quantification. (**B**) Analysis of caspase and poly [ADP-ribose] polymerase (PARP)-1 cleavage by western blot after 24 h of treatment and the corresponding densitometric analysis. **(C-F)** After 8, 24, 48 and 72 h of treatment the type of cell death triggered by TMQ0153 was characterized by flow cytometry (FACS) in presence or absence of the pan-caspase inhibitor z-VAD (50 μM) after Annexin V APC/propidium iodide (PI) staining. Etoposide (Eto; 200 µM, 24 h) was used as a positive control for apoptosis induction. Pictures representative of three independent experiments (top panel) and the corresponding quantification (lower panel). Statistical significance was assessed as *p < 0.05, **p < 0.01, ***p < 0.001 compared to untreated cells. (**G**) Characterization of the type of cell death induced by 50 µM TMQ0153 on K562R by nuclear morphology analysis after 24 and 48 h. Etoposide (Eto; 200 µM, 24 h) was used as a positive control for apoptosis induction. All pictures are representative of three independent experiments and data represent mean (±S.D.) of three independent experiments. Statistical significance was assessed as *p < 0.05, **p < 0.01, ***p < 0.001 compared to untreated cells. **(H)** Characterization of the type of cell death induced by 50 µM TMQ0153 on K562R by FACS after 24h and 48h. Etoposide (Eto; 200 µM, 24 h) was used as a positive control for apoptosis induction. All pictures are representative of three independent experiments and data represent mean (±S.D.) of three independent experiments. Statistical significance was assessed as *p < 0.05, **p < 0.01, ***p < 0.001 compared to untreated cells. Two-way ANOVA (cell viability, microscopy, FACS); post hoc; Sidak’s test. One-way ANOVA (western blot quantification); post hoc; Dunnett’s test. z-VAD: carbobenzoxy-valyl-alanyl-aspartyl-[O-methyl]-fluoromethylketone.

**Suppl. Fig. 6. Effect of Necrostatin-1 on the lower concentration of TMQ0153 on K562 cells. (A)** K562 cells were incubated in presence or absence of 60 µM necrostatin (Nec)-1 for 1 h and 50 μM z-VAD for 1 h before a treatment with the indicated concentrations of TMQ0153, respectively. Etoposide (Eto; 100 µM) was used as a positive control for apoptosis induction. After 24 h of treatment, the type of cell death triggered by TMQ0153 was characterized by flow cytometry (FACS) after Annexin V APC/propidium iodide (PI) staining. Pictures representative of three independent experiments (top panel) and the corresponding quantifications (lower panel) are shown. Statistical significance was assessed as *p < 0.05, **p < 0.01, ***p < 0.001 compared to untreated cells. Two-way ANOVA (FACS); post hoc: Sidak’s test.

**Suppl. Fig. 7.** Basal expression of RIP3 and effect of 5-azacytidine. **(A)** Western blot analysis of lysates from multiple cancer cells lines showing basal RIP3 expression levels (upper panel). Cancer cells were used for comparative studies (β–actin ratios). Quantification of the signals (lower panel). **(B)** K562 cells were pre-treated with 1 µM 5-azacytidine (5-aza) for 3 days and then treated with TMQ0153 for 48 h and cell viability was assessed by Trypan blue assay. RIP3 protein levels were detected by western blot (upper panel) and the corresponding densitometric analysis is shown (lower panel). β–actin was used as loading control. All pictures are representative of three independent experiments and all graphs represent the mean (± S.D.) of three independent experiments. Statistical significance was assessed as *P<0.05, **P<0.01, ***P<0.001 for the indicated comparisons. Two-way ANOVA (cell viability); post hoc: Sidak’s test. One-way ANOVA (western blot quantification); post hoc; Tukey’s test.

**Suppl. Fig. 8. TMQ0153 stimulated autophagy prior to necroptosis.** K562 cells were treated with 30 µM TMQ0153 for the indicated time points. **(A)** Confocal microscopy observations after Hoechst/propidium iodide staining. Arrowhead: intracellular vacuole formation. **(B)** Western blot detection of LC3 protein levels (left panel) and the corresponding densitometric analysis (right panel). 10 µM PP242 for 4 h was used as a positive control for autophagy induction. Statistical significance was assessed as ***p < 0.001 compared to untreated cells unless otherwise specified. One-way ANOVA (western blot); post hoc; Sidak’s test. **(C)** Pictures acquired under light microscopy after Diff-Quik staining (left panel) and the corresponding quantification of the percentage of vacuole-containing cells (right panel). Statistical significance was assessed as *p < 0.05, **p < 0.01 compared to untreated cells unless otherwise specified. One-way ANOVA (diff-quick); post hoc; Dunnett’s test. One-way ANOVA (western blot quantification); post hoc; Sidak’s test.

**Suppl. Fig. 9.** CYBB expression in healthy donors and CML patients from the MILE study. Outliers are represented as dots while healthy donors and CML patients are represented as triangles. Wilcoxon test was used to assess significance.

**Suppl. Fig 10. TMQ0153 induced mitochondrial lesions in K562 cells.** Cells were treated with TMQ0153 for 4h **(A)** and for 8 h **(B)** and mitochondrial morphology was assessed by TEM at 12.000x and 25.000x magniﬁcation. Single arrows and double arrows indicate respectively dilated and giant mitochondria. **(C)** Cells were pre-incubated for 1 h in presence or absence of 60 µM necrostatin (Nec)-1 followed by a treatment with the indicated concentrations of TMQ0153. After 24 h of treatment, MMP was assessed by flow cytometry, respectively. All pictures are representative of three independent experiments and data represent the mean (± S.D.) of three independent experiments. Statistical significance was assessed as *p < 0.05, **p < 0.01, ***p < 0.001 compared to untreated cells unless otherwise specified. One-way ANOVA (mitochondrial membrane); post hoc; Tukey’s test.

**Suppl. Fig. 11. TMQ0153 modulates the redox status of K562 cells. (A)** Reactive oxygen species (ROS) levels were quantified by FACS in K562R cells treated with the indicated concentrations of TMQ0153 and stained with H_2_DCFDA. H_2_O_2_ was used as a positive control for ROS induction. All pictures are representative of three independent experiments and data represent mean (±S.D.) of three independent experiments. All pictures are representative of three independent experiments and data represent mean (±S.D.) of three independent experiments. Statistical significance was assessed as *p < 0.05, **p < 0.01, ***p < 0.001 compared to untreated cells. One-way ANOVA (ROS); post hoc; Tukey’s test. **(B)** Cells were pre-incubated for 1 h in presence or absence of various antioxidants (N-acetyl-L-cysteine (NAC), Tiron and Trolox) and then treated or not with TMQ0153. Reactive oxygen species (ROS) were measured at indicated time points using H_2_DCFDA. H_2_O_2_ was used as a positive control for ROS induction. Data represent the mean (± S.D.) of three independent experiments. Statistical significance was assessed as ***p < 0.001. One-way ANOVA; post hoc; Tukey’s test.

**Suppl. Fig. 12. TMQ0153 treatments released immunogenic cell death markers from K562 cells.** Cells were treated for 24 h with the indicated concentrations of TMQ0153. (**A**) Supernatants from TMQ0153-treated cells were assessed for high mobility group box (HMGB)1 levels. Cells were treated for 24 h with the indicated concentrations of TMQ0153, followed by the analysis of (**B**) extracellular ATP release, **(C)** calreticulin exposure by fluorescence microscopy after 24 h of TMQ0153 treatment at indicated doses, and quantification of calreticulin-positive cells by FACS. (**D**) ectopic ERp57 expression by fluorescence microscopy after 24 h of TMQ0153 treatment at indicated doses and quantification of ERp57-positive cells assessed by FACS. Oxaliplatin (Oxa, 30 µM, 24 h) and shikonin (SHK; 5 µM, 24 h) were used as positive controls for immunogenic cell death induction. All pictures are representative of three independent experiments and data represent the mean (± S.D.) of three independent experiments. Statistical significance was assessed as *p < 0.05, **p < 0.01, ***p < 0.001 compared to untreated cells. One-way ANOVA (HMGB1 assay); post hoc; Tukey’s test. One-way ANOVA (extracellular ATP assay, calreticulin expression, ERp57 expression); post hoc; Dunnett’s test.

**Suppl. Fig.13. Overall mechanism of action of TMQ0153 in CML cells.** (**A**) TMQ0153 regulates apoptosis, necrosis/necroptosis and autophagy *via* a pro-oxidant cellular stress response in CML. The cell death modalities triggered by TMQ0153 are controlled by various factors including energy/ATP availability, the amplification of damages caused by ROS or cellular stress. Depending on the severity of damage and amount of stress encountered by the cells, low doses of TMQ0153 triggered apoptosis, whereas higher concentrations induced necroptosis. TMQ0153-induced autophagy contributes to apoptosis inhibition in favor of a RIP1-dependent necroptotic cell death.
