# Supplementary material for: Tetrahydrobenzimidazole TMQ0153 triggers apoptosis, autophagy and necroptosis crosstalk in chronic myeloid leukemia
Source: Cell Death Dis. 2020 Feb 7;11(2):109. doi: 10.1038/s41419-020-2304-8 (PMC7007439; doi:10.1038/s41419-020-2304-8)
Supplement: Supplementary file 2 — Supplementary tables [file 41419_2020_2304_MOESM2_ESM.docx]

**Supplementary Tables**

**Tetrahydrobenzimidazole TMQ0153 triggers apoptosis, autophagy and necroptosis crosstalk in chronic myeloid leukemia**

**Song et al.**

**Supplementary Table I: *In silico* prediction for the drug-likeness of TMQ0153 compared to hydroquinone based on Lipinski’s ‘rule of five’** [^61^](#_ENREF_61).

| **Drug-likeness parameter** | **Values** | | |
| --- | --- | --- | --- |
|  | **Theoretical** | **TMQ0153** | **Hydroquinone** |
| **MW (kDa)*** | **180 ≤ x ≤ 500** | 276.5 | 110 |
| **Hydrogen bond donors** | **≤ 5** | 1 | 2 |
| **Hydrogen bond acceptors** | **≤ 10** | 4 | 2 |
| **LogP (lipophilicity)** | **≤ 5** | 2.16 | 1.10 |
| **Molar refractivity** | **40 ≤ x ≤ 130** | 74.5 | 29.8 |

*MW: molecular weight

**Supplementary Table II: Effect of TMQ0153 on human cancer cell viability.**

| **Cancer model** | | **IC_50 (_µM)*** | | | |
| --- | --- | --- | --- | --- | --- |
| **Cancer type** | **Cell line** | **8 h** | **24 h** | **48 h** | **72 h** |
| **Blood** | **K562** | > 50 | 35.4 ± 0.6 | 28.7 ± 0.7 | 26.1 ± 0.7 |
|  | **K562R** | > 50 | > 50 | > 50 | 43.4 ± 1.8 |
|  | **KBM5** | > 50 | > 50 | 20.0 ± 2.3 | 14.5 ± 0.3 |
|  | **KBM5R** | > 50 | > 50 | 20.7 ± 2.7 | 15.1 ± 0.8 |
|  | **MEG01** | > 50 | > 50 | 45.3 ± 2.7 | 28.8 ± 1.7 |
| **Lung** | **A549** | > 50 | > 50 | > 50 | > 50 |
| **Prostate** | **PC3** | > 50 | > 50 | > 50 | 49.0 ± 0.9 |
| **Breast** | **MCF7** | > 50 | > 50 | > 50 | 46.7 ± 2.4 |

*IC50 values were calculated on data obtained from Trypan blue assays and represent the mean ± S.D. of three independent experiments.

R: Imatinib-resistant.**Supplementary Table III: Differential toxicity of TMQ0153 on PBMCs vs. K562.**

|  | | **Fold change** | | |
| --- | --- | --- | --- | --- |
| **Concentration (µM)** | **24 h** | | **48 h** | **72 h** |
| **0** | 1.00 | | 1.00 | 1.00 |
| **10** | 0.99 | | 0.87 | 0.94 |
| **20** | 1.06 | | 0.87 | 0.81 |
| **30** | 1.25 | | 1.27 | 1.81 |
| **40** | 1.73 | | 2.33 | 6.77 |
| **50** | 2.27 | | 13.12 | 149.24 |
