# Supplementary figures and images for: Tetrahydrobenzimidazole TMQ0153 triggers apoptosis, autophagy and necroptosis crosstalk in chronic myeloid leukemia
Source: Cell Death Dis. 2020 Feb 7;11(2):109. doi: 10.1038/s41419-020-2304-8 (PMC7007439; doi:10.1038/s41419-020-2304-8)

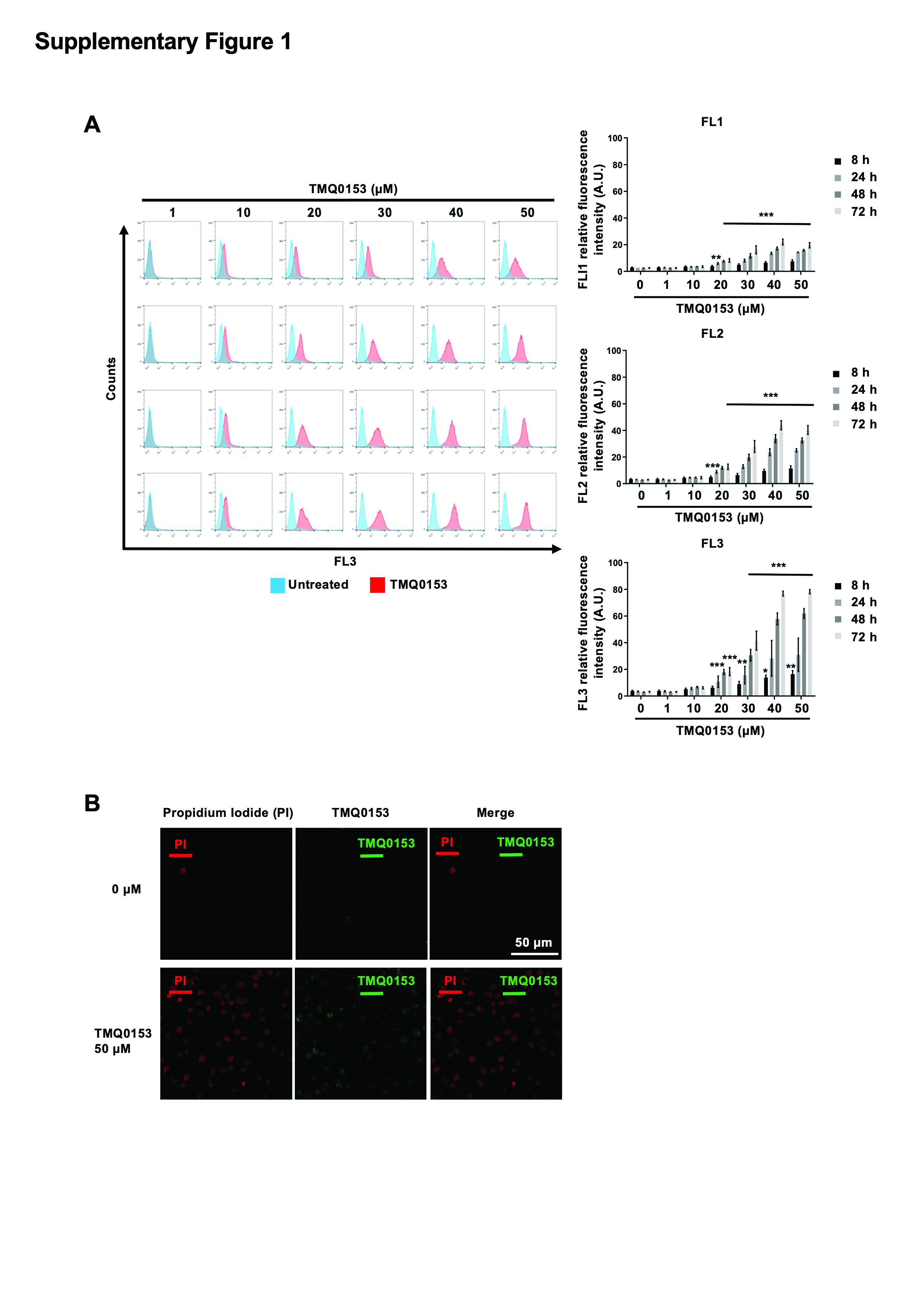

Supplement: Supplementary file 3 — Supplementary figure 1 [file 41419_2020_2304_MOESM3_ESM.tif]

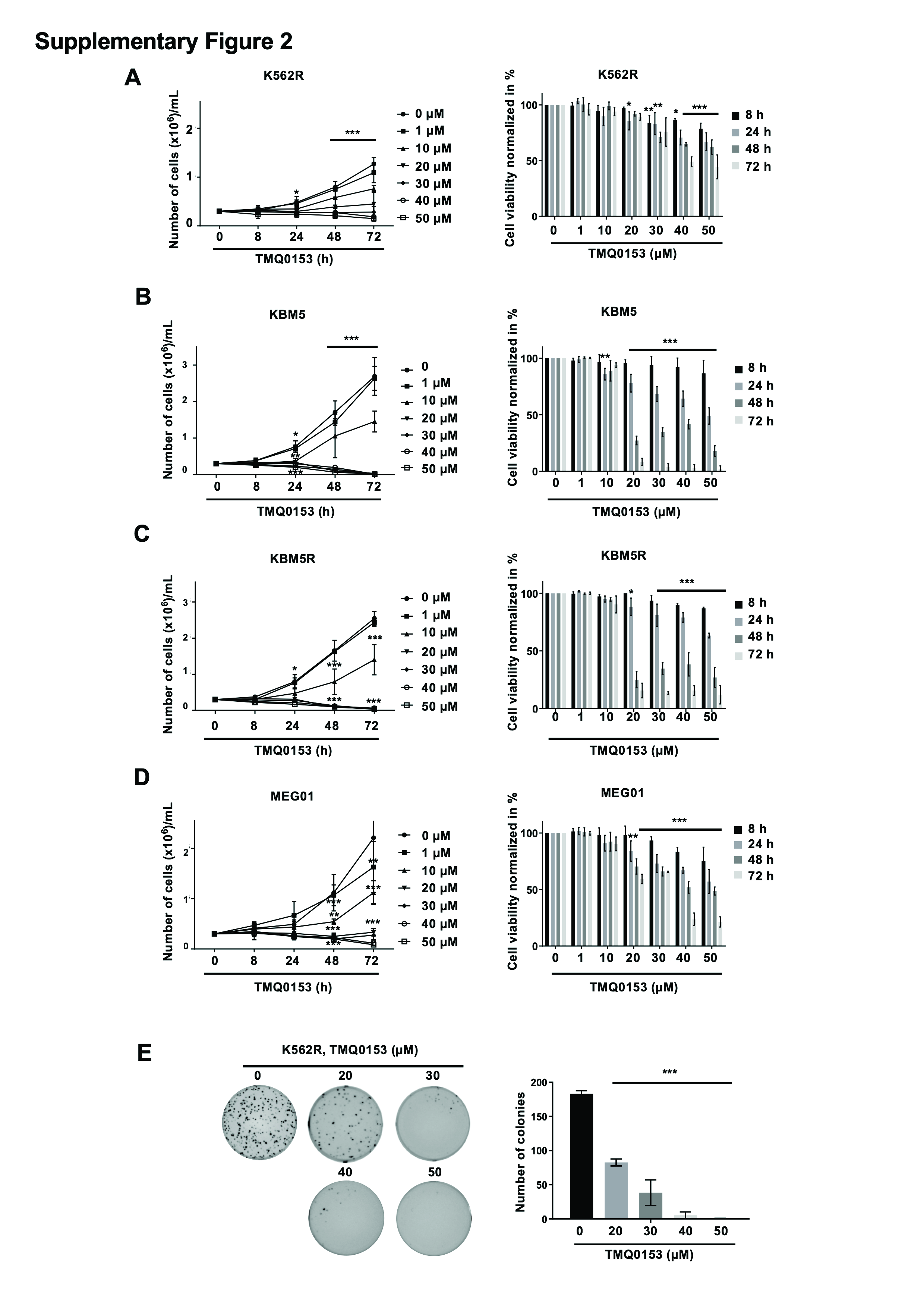

Supplement: Supplementary file 4 — Supplementary figure 2 [file 41419_2020_2304_MOESM4_ESM.tif]

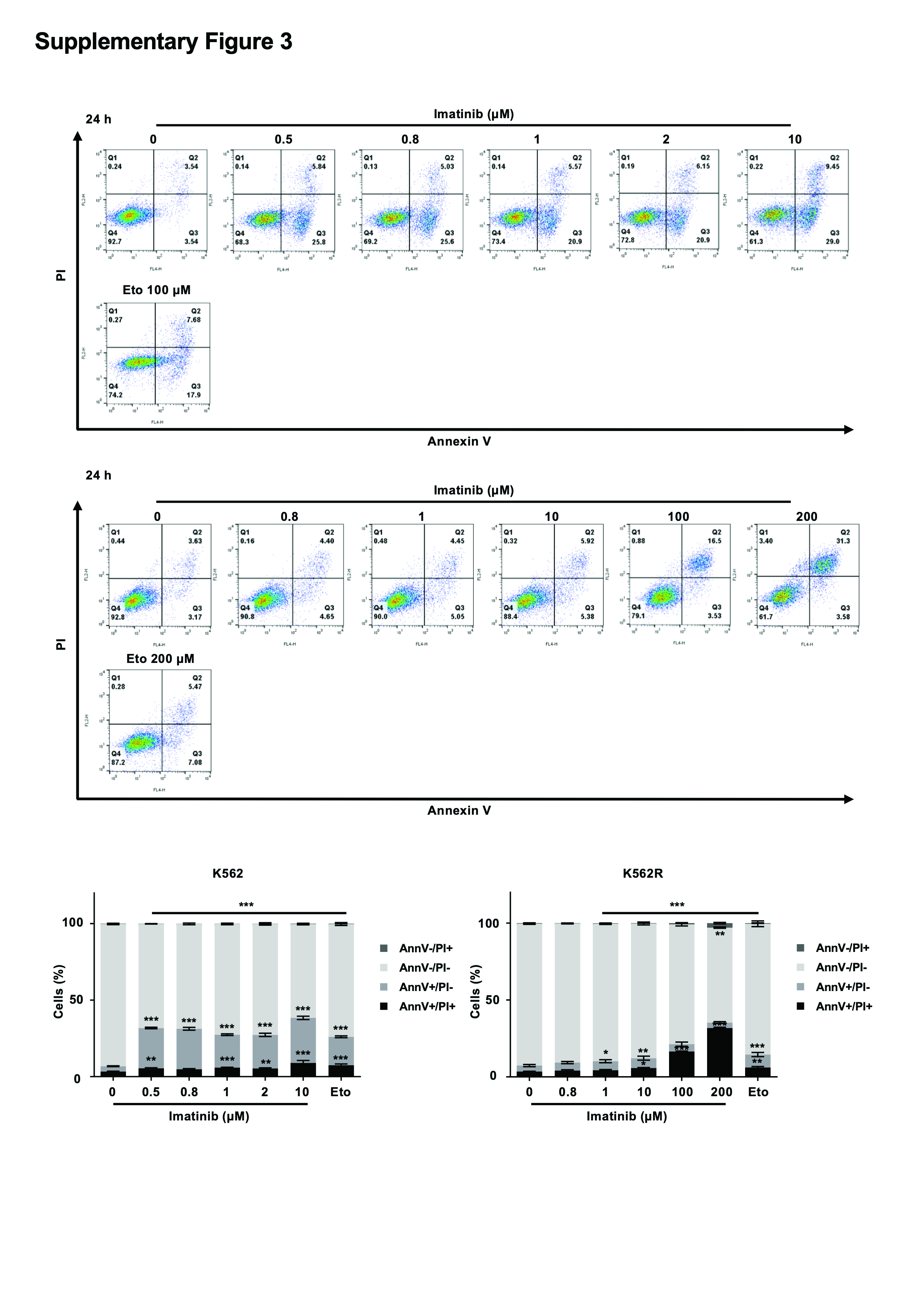

Supplement: Supplementary file 5 — Supplementary figure 3 [file 41419_2020_2304_MOESM5_ESM.tif]

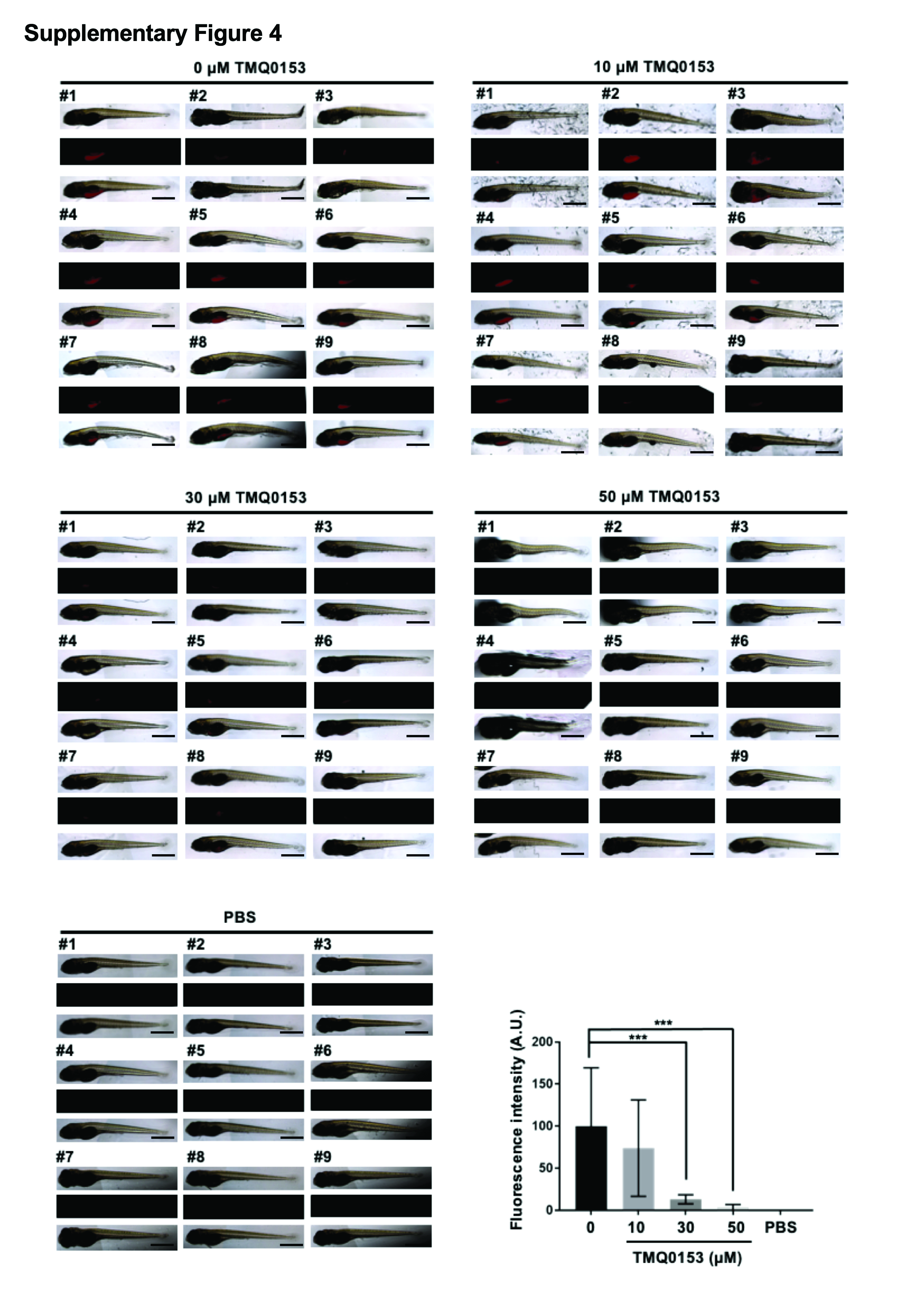

Supplement: Supplementary file 6 — Supplementary figure 4 [file 41419_2020_2304_MOESM6_ESM.tif]

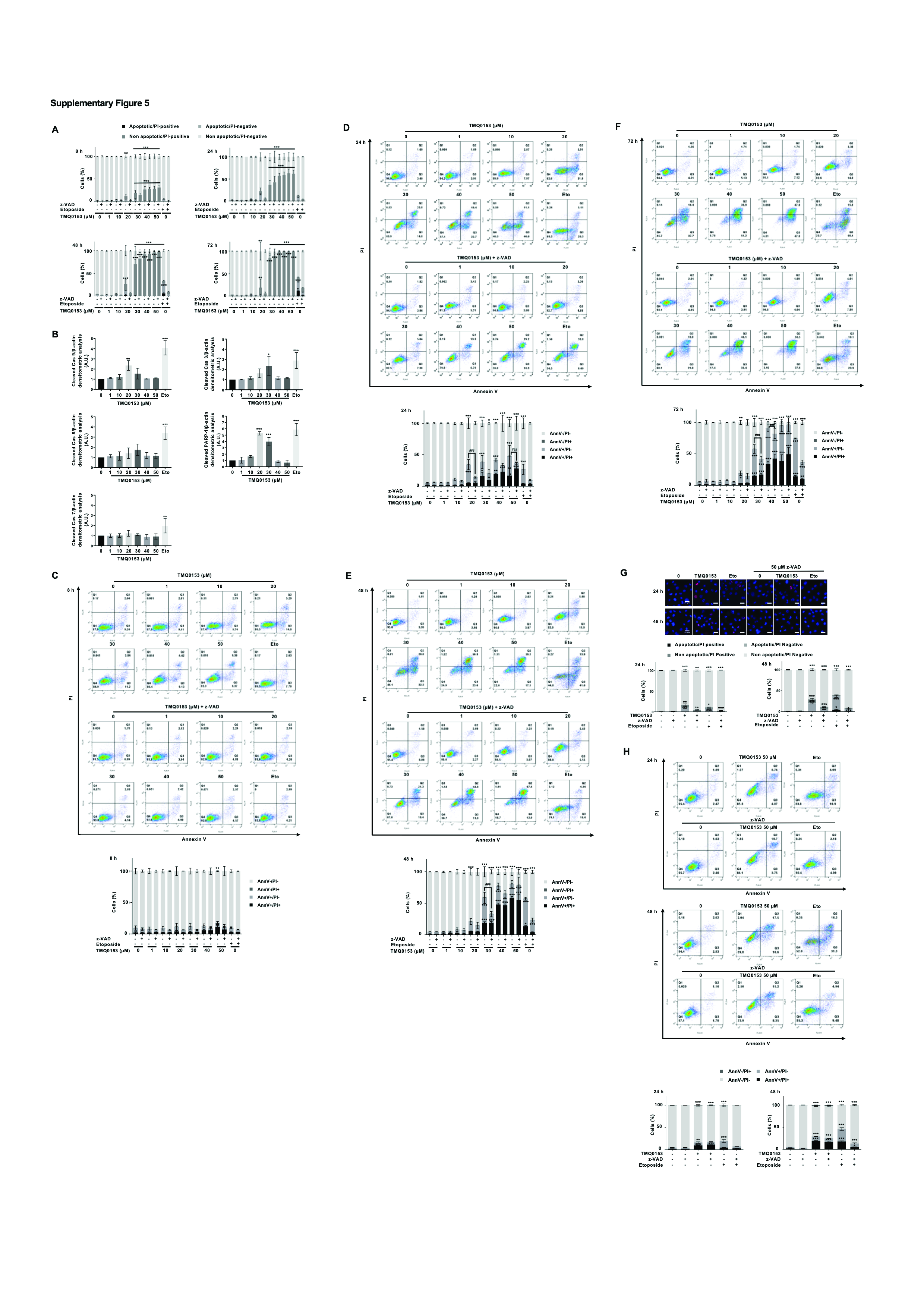

Supplement: Supplementary file 7 — Supplementary figure 5 [file 41419_2020_2304_MOESM7_ESM.tif]

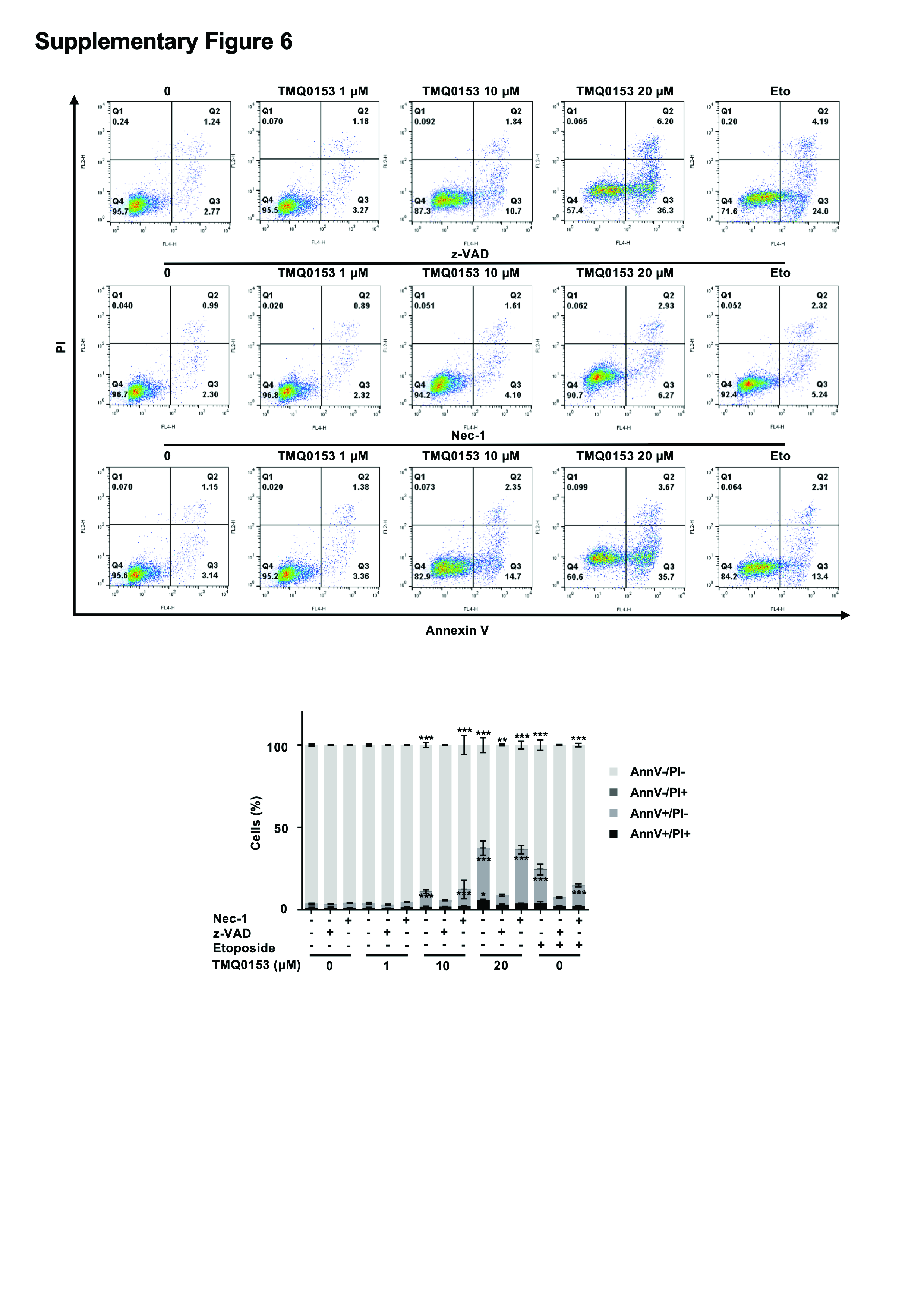

Supplement: Supplementary file 8 — Supplementary figure 6 [file 41419_2020_2304_MOESM8_ESM.tif]

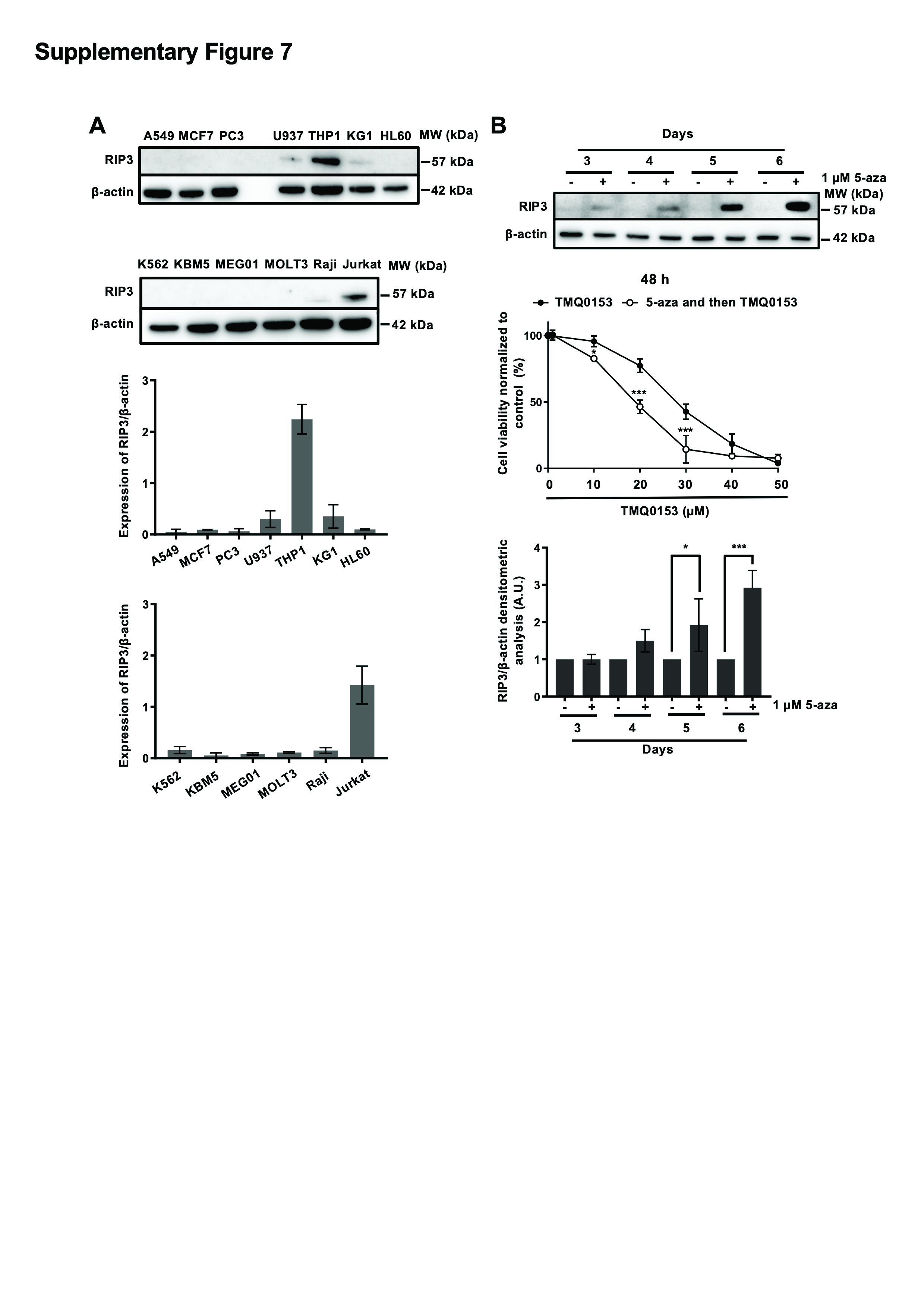

Supplement: Supplementary file 9 — Supplementary figure 7 [file 41419_2020_2304_MOESM9_ESM.tif]

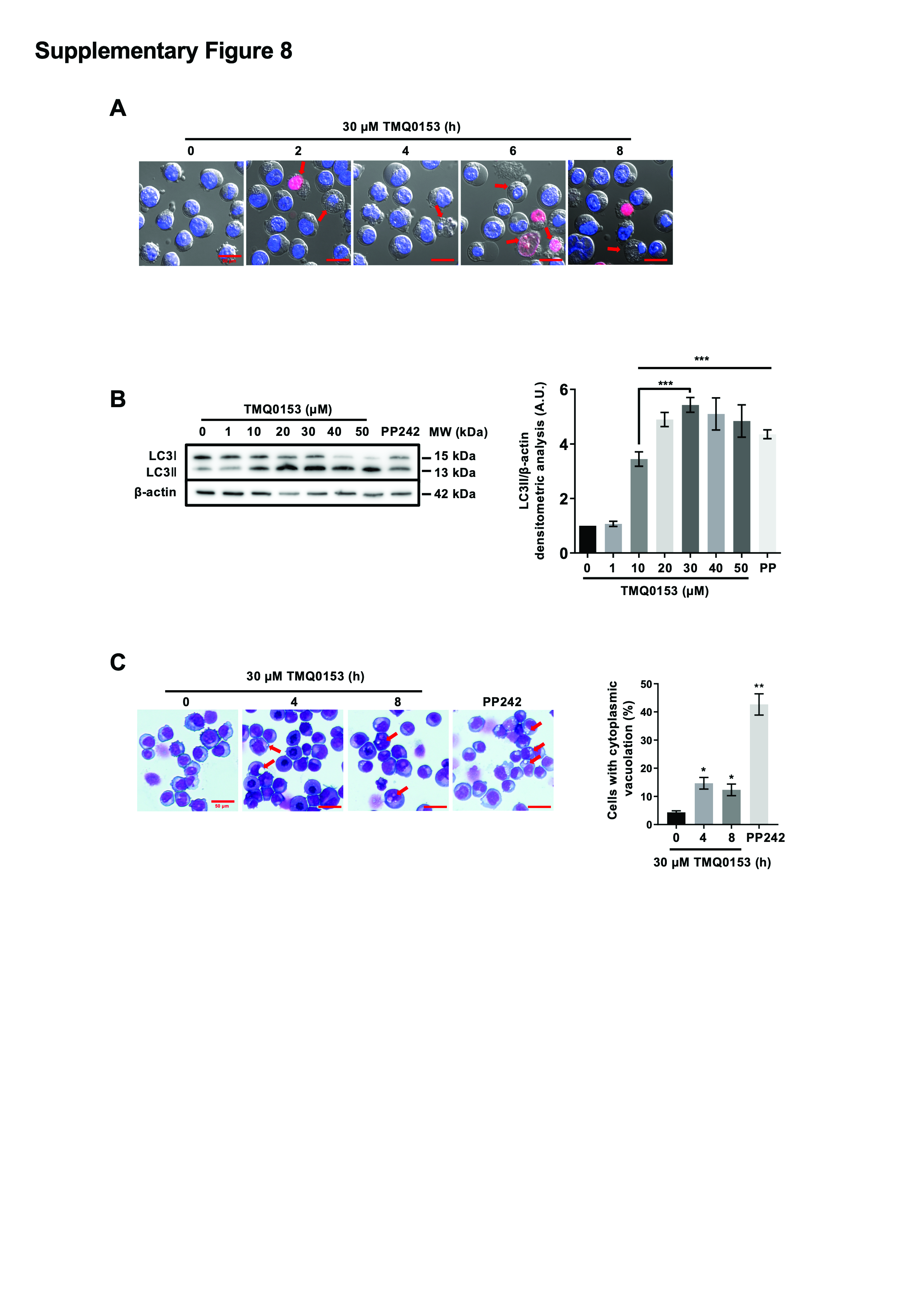

Supplement: Supplementary file 10 — Supplementary figure 8 [file 41419_2020_2304_MOESM10_ESM.tif]

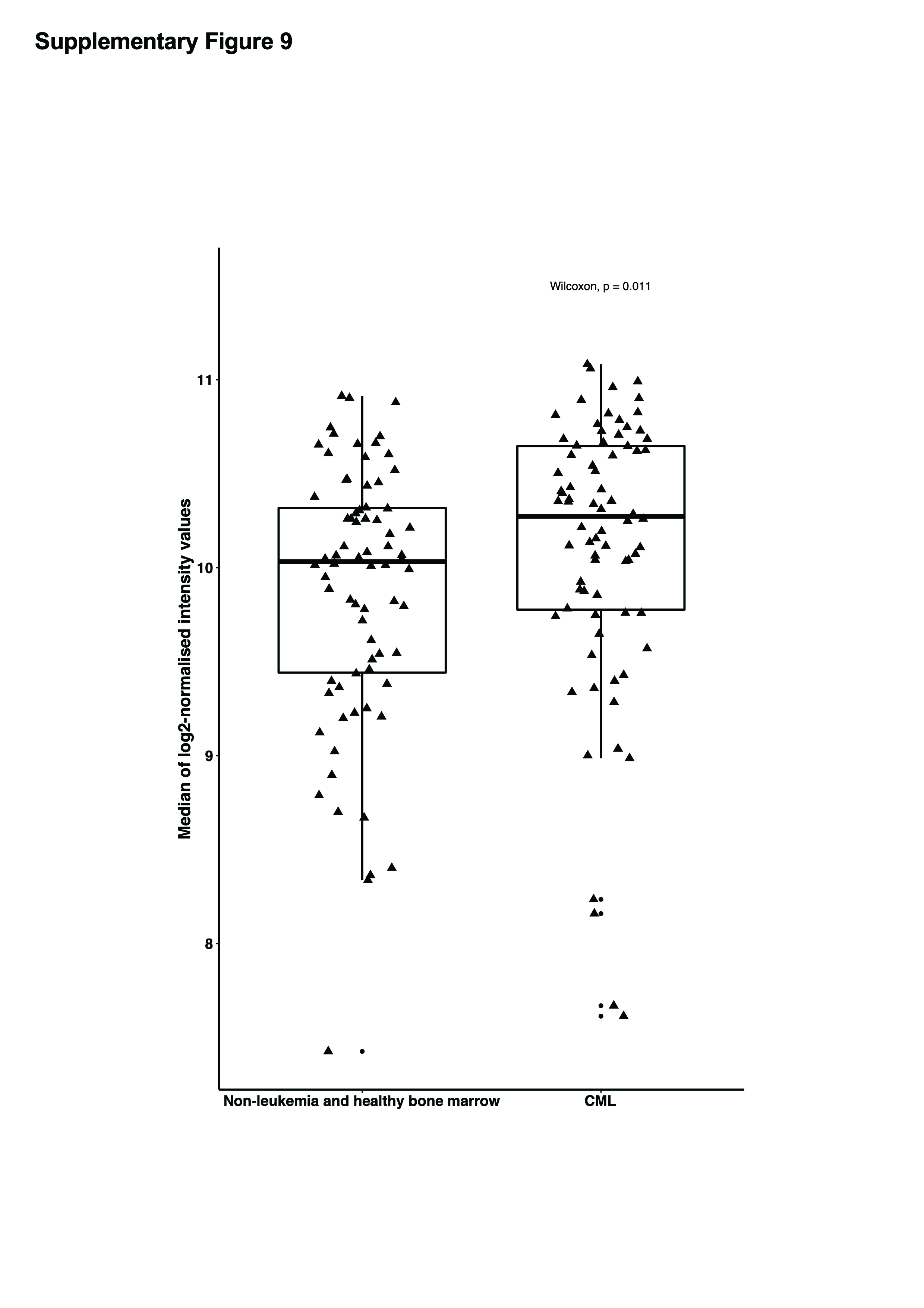

Supplement: Supplementary file 11 — Supplementary figure 9 [file 41419_2020_2304_MOESM11_ESM.tif]

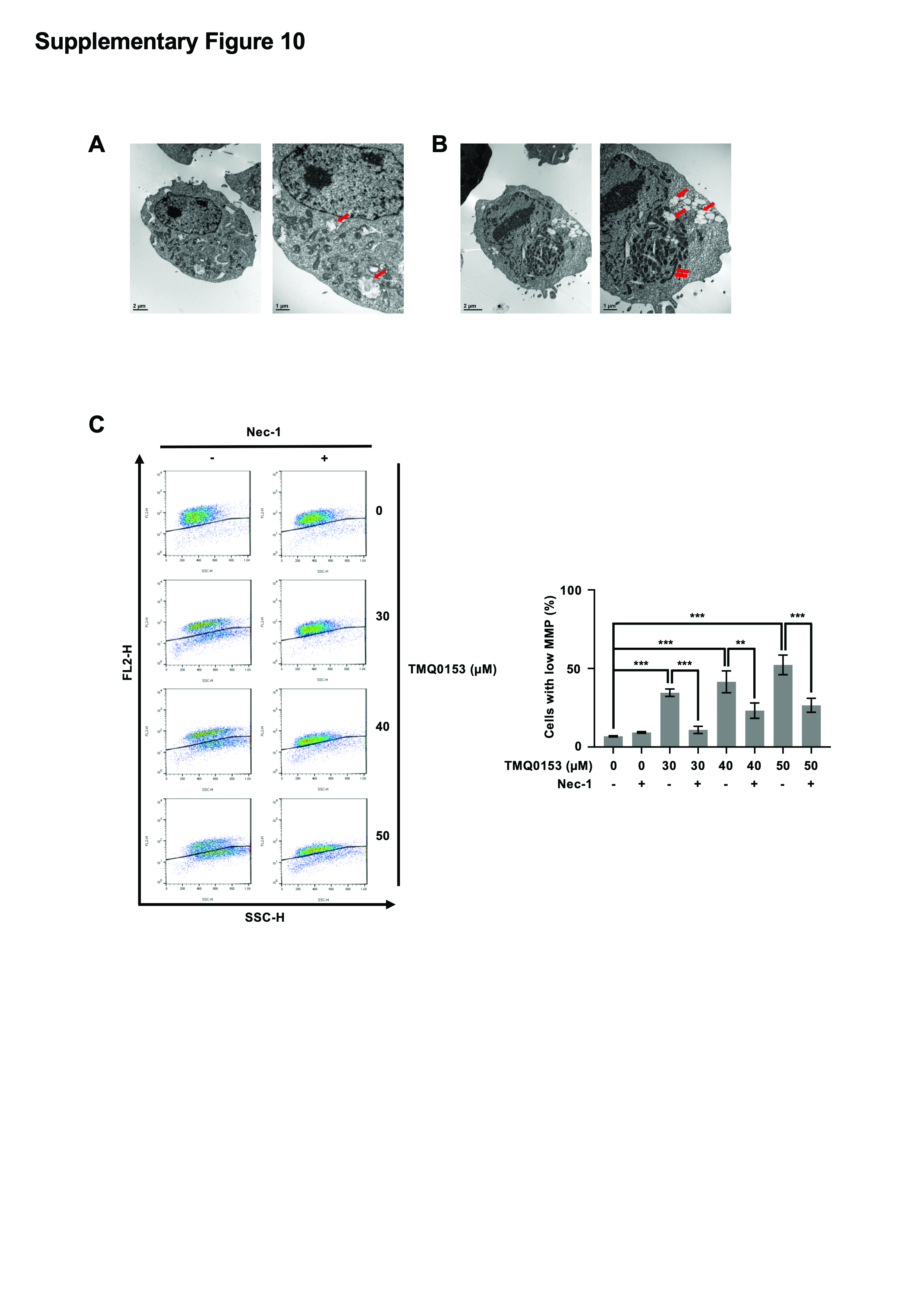

Supplement: Supplementary file 12 — Supplementary figure 10 [file 41419_2020_2304_MOESM12_ESM.tif]

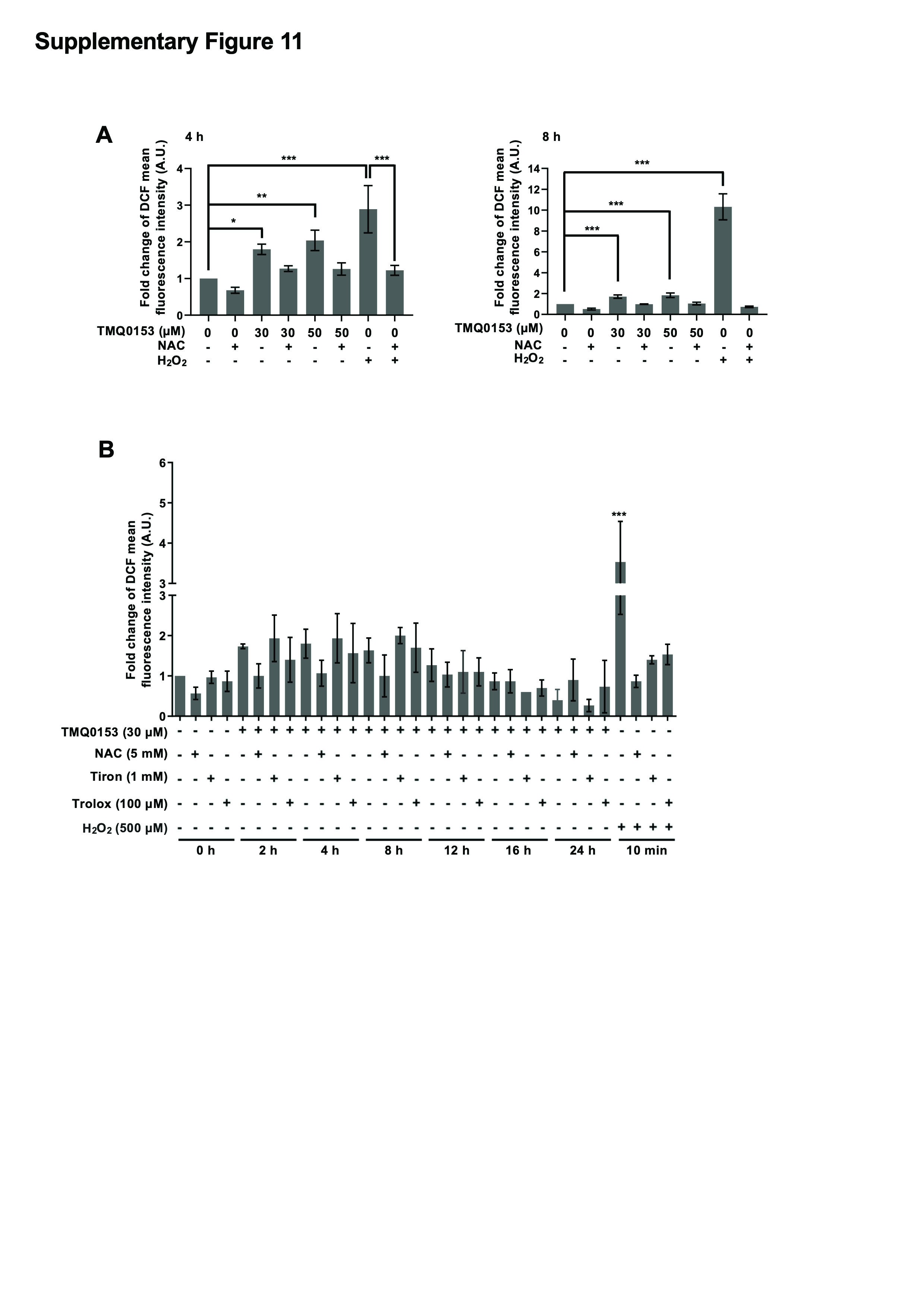

Supplement: Supplementary file 13 — Supplementary figure 11 [file 41419_2020_2304_MOESM13_ESM.tif]

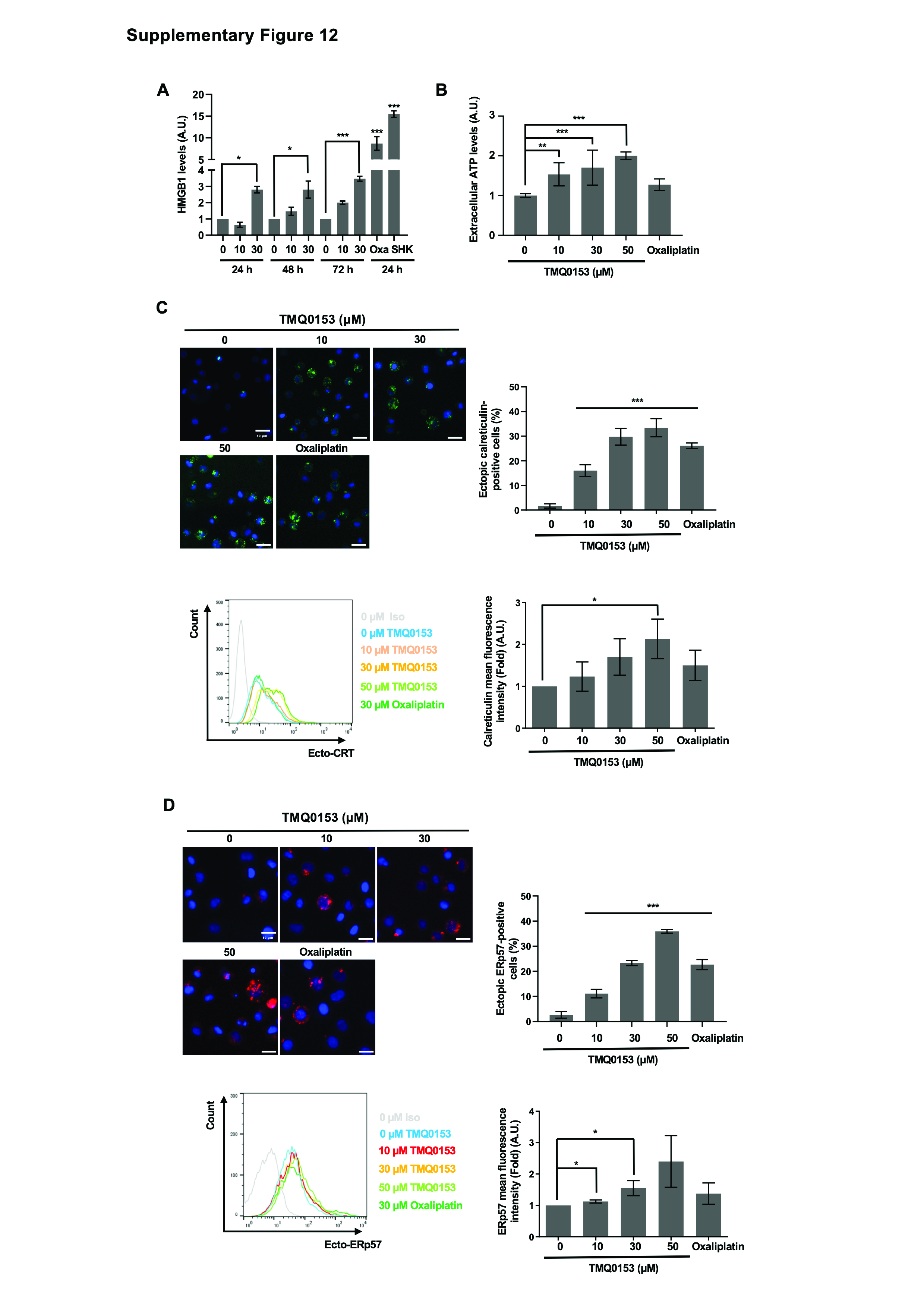

Supplement: Supplementary file 14 — Supplementary figure 12 [file 41419_2020_2304_MOESM14_ESM.tif]

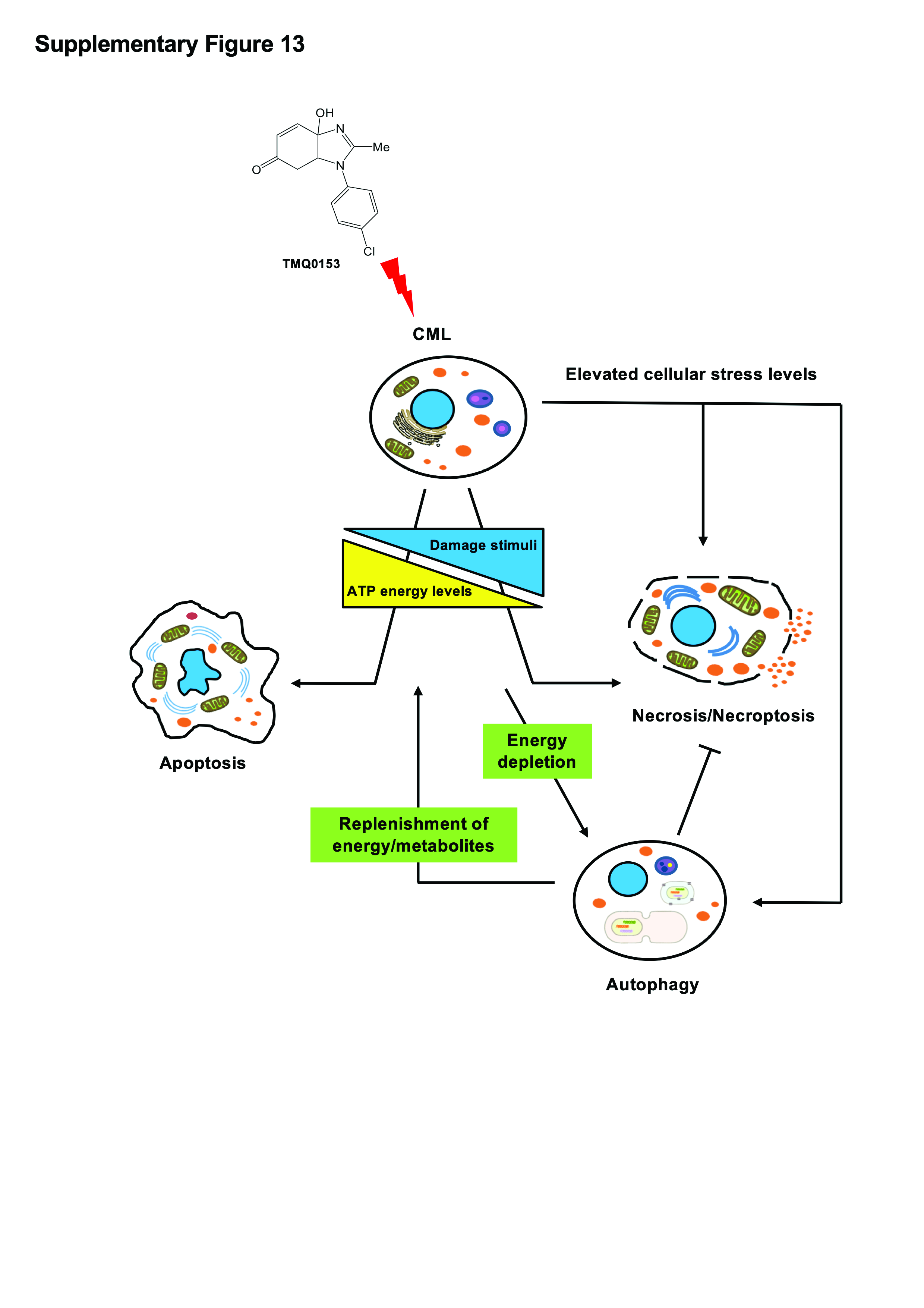

Supplement: Supplementary file 15 — Supplementary figure 13 [file 41419_2020_2304_MOESM15_ESM.tif]
